# Antagonistic network signature of motor function in Parkinson’s disease revealed by connectome-based predictive modeling

**DOI:** 10.1038/s41531-022-00315-w

**Published:** 2022-04-22

**Authors:** Xuyang Wang, Kwangsun Yoo, Huafu Chen, Ting Zou, Hongyu Wang, Qing Gao, Li Meng, Xiaofei Hu, Rong Li

**Affiliations:** 1 The Clinical Hospital of Chengdu Brain Science Institute, MOE Key Laboratory for Neuroinformation, High-Field Magnetic Resonance Brain Imaging Key Laboratory of Sichuan Province, School of Life Science and Technology, University of Electronic Science and Technology of China, Chengdu, 610054 People’s Republic of China; 2 Department of Psychology, Yale University, New Haven, CT 06520 USA; 3 School of Mathematical Sciences, University of Electronic Science and Technology of China, Chengdu, 610054 People’s Republic of China; 4 Department of Radiology, Xiangya Hospital, Central South University, Changsha, 410008 People’s Republic of China; 5 Department of Radiology, Southwest Hospital, Third Military Medical University, Chongqing, 610054 People’s Republic of China

**Keywords:** Parkinson's disease, Network models

## Abstract

Motor impairment is a core clinical feature of Parkinson’s disease (PD). Although the decoupled brain connectivity has been widely reported in previous neuroimaging studies, how the functional connectome is involved in motor dysfunction has not been well elucidated in PD patients. Here we developed a distributed brain signature by predicting clinical motor scores of PD patients across multicenter datasets (total *n* = 236). We decomposed the Pearson’s correlation into accordance and discordance via a temporal discrete procedure, which can capture coupling and anti-coupling respectively. Using different profiles of functional connectivity, we trained candidate predictive models and tested them on independent and heterogeneous PD samples. We showed that the antagonistic model measured by discordance had the best sensitivity and generalizability in all validations and it was dubbed as Parkinson’s antagonistic motor signature (PAMS). The PAMS was dominated by the subcortical, somatomotor, visual, cerebellum, default-mode, and frontoparietal networks, and the motor-visual stream accounted for the most part of predictive weights among network pairs. Additional stage-specific analysis showed that the predicted scores generated from the antagonistic model tended to be higher than the observed scores in the early course of PD, indicating that the functional signature may vary more sensitively with the neurodegenerative process than clinical behaviors. Together, these findings suggest that motor dysfunction of PD is represented as antagonistic interactions within multi-level brain systems. The signature shows great potential in the early motor evaluation and developing new therapeutic approaches for PD in the clinical realm.

## Introduction

Parkinson’s disease (PD) is a common neurodegenerative disease affecting millions of people worldwide, especially in terms of daily motor behaviors^1^. Pathologically, the well-known hallmark of PD is the degeneration of nigrostriatal dopaminergic neurons and the presence of α-synuclein-containing Lewy bodies^2,3^. The initial abnormality seems to trigger widespread aberrant neuronal activities and signaling pathways that lead to the decline of neurological functions, such as impaired motor function in PD^3–5^. Although striatal dopamine loss induces motor dysfunction, this single perspective gives a restricted clinical picture and limits potential therapeutic approaches^6^. Thus, probing motor dysfunction in PD from a distributed network perspective has a potential to provide an overarching framework and new insights into the neural substrates of motor-enabled factors.

From a network perspective, appropriate behaviors rely on not only the functionally specialized brain regions but also the cooperative interactions among distributed entities^7^. Functional connectivity (FC), one of the most well-known synchronized phenomena, is usually measured noninvasively with resting-state functional MRI (rs-fMRI). With the advantages of high spatial resolution to cover the whole brain and easily performing, rs-fMRI provides a vehicle to investigate FC patterns on different populations^8–10^. In the context of PD, neuroimaging evidence has converged to reveal the deterioration of functional interactions in the cortico-striatum circuitry^5,11,12^ which is also associated with motor impairment clinically assessed by the third part of Unified Parkinson’s Disease Rating Scale (UPDRS III)^13^. For example, Szewczyk-Krolikowski et al.^14^ reported the reduced connectivity between the basal ganglia network and widespread frontal, temporal, and parietal cortices in PD patients compared with healthy controls. Another study by Campbell et al.^15^ found that patients with PD had significantly reduced sensorimotor FC, which correlated with the reduced cerebrospinal fluid (CSF) levels of α-synuclein. A classical opinion related to this phenomenon is that neurodegeneration can disrupt the highly synchronized spontaneous blood oxygen level-dependent (BOLD) fluctuations^16,17^. Although previous studies have revealed that PD is associated with network changes in distributed brain areas, the statistical inferences from group-level analyses cannot evaluate individual clinical behaviors^18^. There is still a gap in understanding how the human connectome shape motor performance at the single-subject level.

Predictive modeling has recently emerged as a powerful tool to discover neural substrates of human cognition and evaluate individual behaviors quantitively^19^. Prior studies have demonstrated that functional connectomes can be stable across multiple days, which are dominated by common organizational principles and individual features^20,21^. Based on the connectome-based predictive modeling (CPM)^22^, numerous studies have decoded human characteristics from functional interactions^23–25^ and revealed neural architectures of specific brain states^26,27^. To date, the most popular measure of FC is Pearson’s correlation. Despite its usefulness in previous rs-fMRI studies, this statistical dependency is coarse-grained which ignores point to point effects between two time-varying series. These challenges can be addressed with recently proposed measures of FC—discordance and accordance^28^, which quantify out-of-phase anti-correlation and in-phase synchronization respectively. In the context of PD, previous studies have consistently demonstrated that FC was reduced among brain networks^12,14,15,29,30^, which seems to be a result of antagonistic effects induced by dopamine depletion. Accordingly, the predictive model based on discordance connectivity has the potential to provide more insights into the mapping between brain systems and clinical motor behaviors in PD.

Here, we aimed to represent the motor signature of PD using the CPM across multicenter dataset (*n* = 236). First, we constructed candidate predictive models with discordance, accordance, and Pearson’s correlation on the discovery cohort and quantified the sensitivity of models by cross validation (Study 1, n1 = 71). Then, the models with fixed parameters, which derived from the whole discovery cohort, were applied to three independent samples (Study 2, n2 = 45; Study 3, n3 = 60; Study 4, n4 = 60) to validate the generalizability. We hypothesized that although the candidate models would share some common brain representations of motor function, the predictive model profiled with discordance might show better sensitivity and generalizability. In addition, we expected the predictive model might have different patterns along with the disease course given the progressive nature of PD. Thus, we further investigated the relationship between the predictive residuals and disease stages in PD.

## Results

### Subjects enrollment

We enrolled a total of 236 patients with PD enrolled from nine sites to construct and validate connectome-based predictive models for motor function in this work (from Study 1 to Study 4). The dataset from Southwest Hospital of the Third Military Medical University sample (TMMU, *n* = 116) was divided into two samples (Study 1 and Study 2). Study 1 (*n* = 71) served as a discovery cohort to establish candidate predictive models based on different profiles of FC and to quantify their sensitivity. Study 2 consisted of subjects excluded from Study 1 due to large head movements (*n* = 45) to test the generalizability of predictive models. Study 3 served as an external cohort (*n* = 60) collected with a different scanner at Xiangya Hospital of Central South University (CSU) to validate the generalizability out of a single center. Study 4 consisted of 60 subjects (mainly covered the white race) collected from the Parkinson’s Progression Markers Initiative (https://www.ppmi-info.org, PPMI) to test the generalizability out of one ethnicity. In brief, PPMI is a public, intranational, and multicenter dataset which can be used to identify the biomarkers of PD progression^31^. Notably, subjects used in Study 4 were collected from seven centers and were scanned with the same scanner (Siemens) and parameters. Finally, the predictive model with the best sensitivity and generalizability (termed as the final predictive model) was used to defined a brain signature of motor function in PD through all studies.

### Functional connectome-based predictive models for motor function in PD

**Fig. 1:**
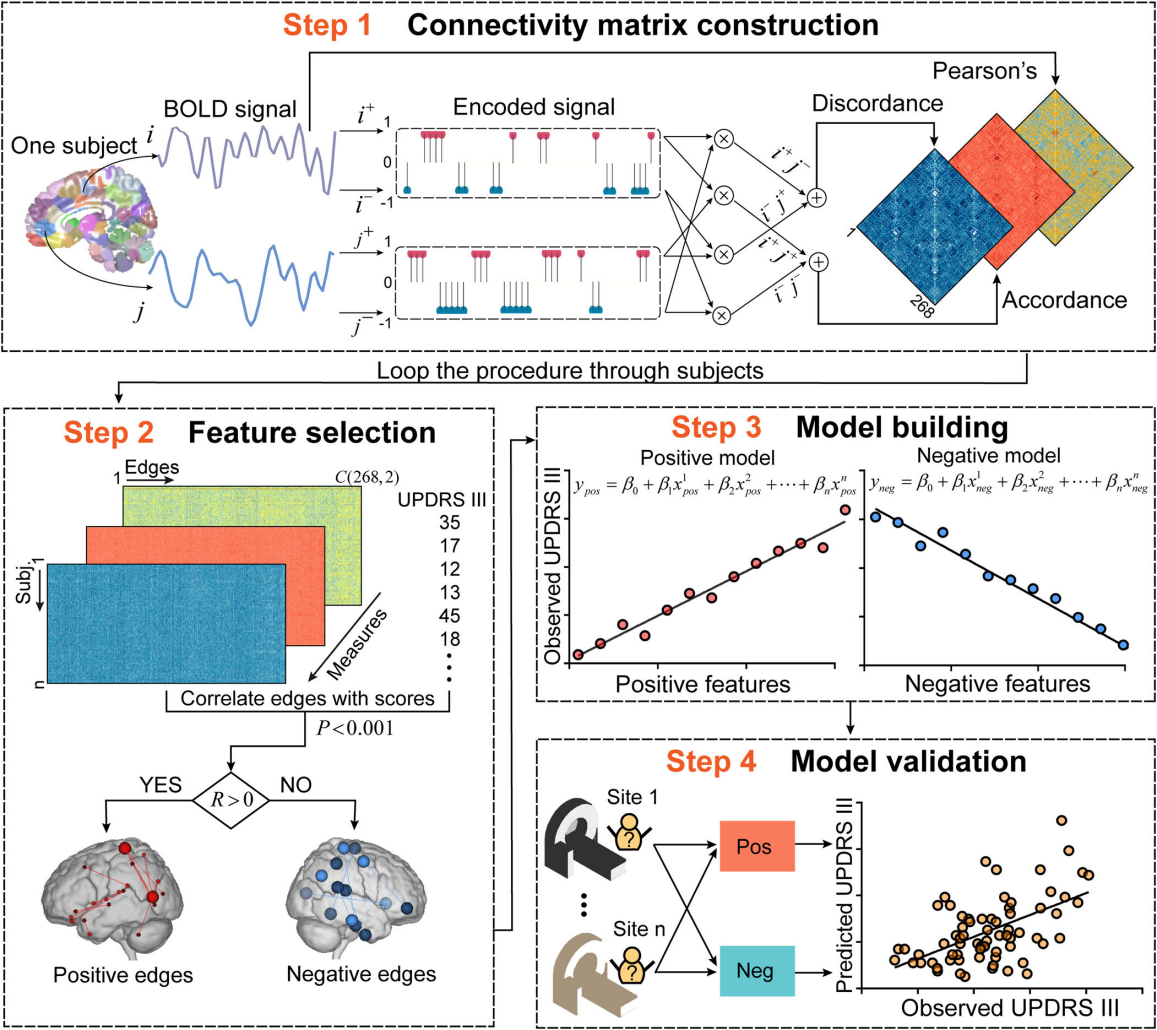
Schematic representation of connectome-based predictive modeling for motor dysfunction in PD. Step 1: We extracted the mean signal from regions of interest (ROIs), then positive extreme values of the z-transformed time series were encoded as 1, whereas negative extreme values were encoded as -1. The encoded series was subsequently sent into two layers. The first layer (dot product layer) produced sub-states of connectivity for each pair of ROIs, and the last layer (accumulation layer) quantified the synchronized interactions (accordance) and antagonistic interactions (discordance). Connectivity matrices based on three FC measures were constructed for each subject. Step 2: We looped step 1 through subjects and created a group-level matrix (*m*×*n*, where m represents subjects, and n represents FC). The significantly motor-correlated edges (*P* < 0.001) were selected and divided into positive features (*R* > 0) and negative features (*R* < 0). Step 3: We trained a multivariate linear regression model using partial least squares based on selected features. Step 4: Apply models to novel subjects in independent samples to assess the generalizability. Brain images in this figure were obtained from the Bioimage Suite (https://bioimagesuiteweb.github.io/webapp/), which is an open source software package. UPDRS III Unified Parkinson’s Disease Rating Scale motor examination; FC functional connectivity.

**Fig. 2:**
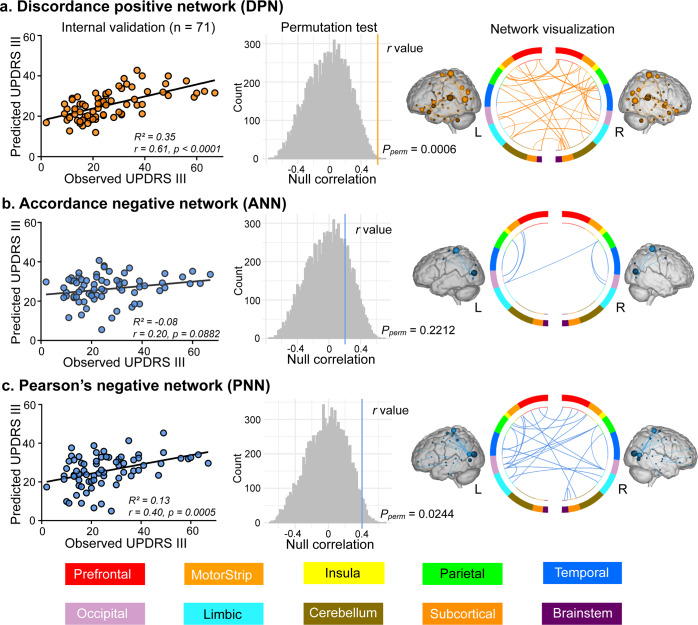
Internal validation of predictive models and network visualization. Using the LOOCV method to obtain predicted scores, the evaluation of predictive performance was based on (1) the Pearson’s correlation between observed UPDRS III scores and predicted UPDRS III scores, and (2) the predicted *R*^*2*^ (the left column). Permutation test was performed by comparing true *r* values (colored vertical lines in the middle column) with a null distribution of *r* values, yielding a significant effect for DPN and PNN models. Edges with significant (*P* < 0.001) and robust (exceed 95% of iterations) dependency on UPDRS III scores were reserved in the predictive networks (the right column). The network visualization was completed with Bioimage Suite (https://bioimagesuiteweb.github.io/webapp/). The color bars at the bottom of this figure represent which brain parcellation the nodes are assigned to on the basis of a lobe scheme. L left hemisphere, R right hemisphere, LOOCV leave-one-out cross-validation, DPN discordance positive network, PNN Pearson’s negative network.

In Study 1 (*n* = 71), we established candidate predictive models with the three profiles of FC (Pearson’s correlation, accordance, and discordance; shown in Fig. 1). According to the protocol of CPM^22^, we first demonstrated that UPDRS III scores were not correlated with head motions in all studies (Study 1: *r*_(69)_ = −0.12, *P* = 0.3278; Study 2: *r*_(43)_ = −0.13, *P* = 0.3979; Study 3: *r*_(58)_ = −0.03, *P* = 0.8279; Study 4: *r*_(58)_ = −0.03, *P* = 0.7989). During the feature selection step in Fig. 1, the input connectome had 35,778 distinct edges per subject. To reduce the dimensions of connectome for further brain-behavior modeling, partial correlation (*P* < 0.001) was used to minimize the confounding effect of age. Three candidate predictive models were then defined according to the profiles of FC and their relationship with UPDRS III scores, resulting in (1) discordance positive network (discordance connectivity correlated with UPDRS III scores positively, DPN), (2) accordance negative network (accordance connectivity correlated with UPDRS III scores negatively, ANN), and (3) Pearson’s negative network (Pearson’s connectivity correlated with UPDRS III scores negatively, PNN) models. We observed that the DPN had the best sensitivity in Study 1 (prediction *R*^2^ = 0.35, *r* = 0.61, *P*_permutation_ = 0.0006, Fig. 2a). While PNN could also predict observed scores to a certain degree (prediction *R*^2^ = 0.13, *r* = 0.40, *P*_permutation_ = 0.0244, Fig. 2c), ANN showed the worst performance (prediction *R*^*2*^ = − 0.08, *r* = 0.20, *P*_permutation_ = 0.2212, Fig. 2b). Statistical significances in this internal validation were approved by Steiger’s z test (DPN vs PNN: *z* value = 2.52, *P* = 0.0057; DPN vs ANN: *z* value =3.73, *P* = 0.0001). The other three models (DNN, APN, and PPN) were abandoned in further external validations due to the relatively poor predictive performance in Study 1 (Supplementary Fig. 1). For network visualization (shown as the third column of Fig. 2), we retained connections that appeared at least 95% times in all iterations using the leave-one-out cross-validation (LOOCV) method. Finally, there were 33 edges in DPN, 6 edges in ANN, and 22 edges in PNN. Information about each edge was listed in Supplementary Table 1. Results of other thresholds (90% and 100%) are also depicted in Supplementary Fig. 2.

CPM analysis with different parameters (*q* ranged from 0.8 to 0.9 with a step of 0.01; *P* ranged from 0.001 to 0.01 with a step of 0.001) was repeated to test the stability of predictive performance (threshold *q* for binarization and threshold *p* for feature selection). All predicted *r* values between the observed scores and predicted scores are displayed in Supplementary Fig. 3. This result indicated that DPN, ANN, and PNN had a more robust and stronger predictive performance than their corresponding opposite modes (DNN, APN, and PAN; Supplementary Fig. 4a–c). Moreover, a paired sample t test revealed that the predictive performance of DPN was significantly better than ANN (*t*_(109)_ = 2.80, *P* = 0.0061, Supplementary Fig. 4d). We also examined the predictive performance of DPN on Shen-368 atlas^32^, Power-264 atlas^33^, and Fan-246 atlas^34^ to explore the influence of different brain parcellations. By setting the *P* value at 0.001 and ranging *q* value from 0.8 to 0.9, we repeated CPM on each brain atlas and computed the correlation between predicted scores and observed scores (Supplementary Fig. 5). This analysis revealed that DPN could keep a relatively consistent predictive performance across different brain parcellation schemes. All results in this part illustrated the reliable predictive performance of DPN.

### Network-level representations of predictive models

**Fig. 3:**
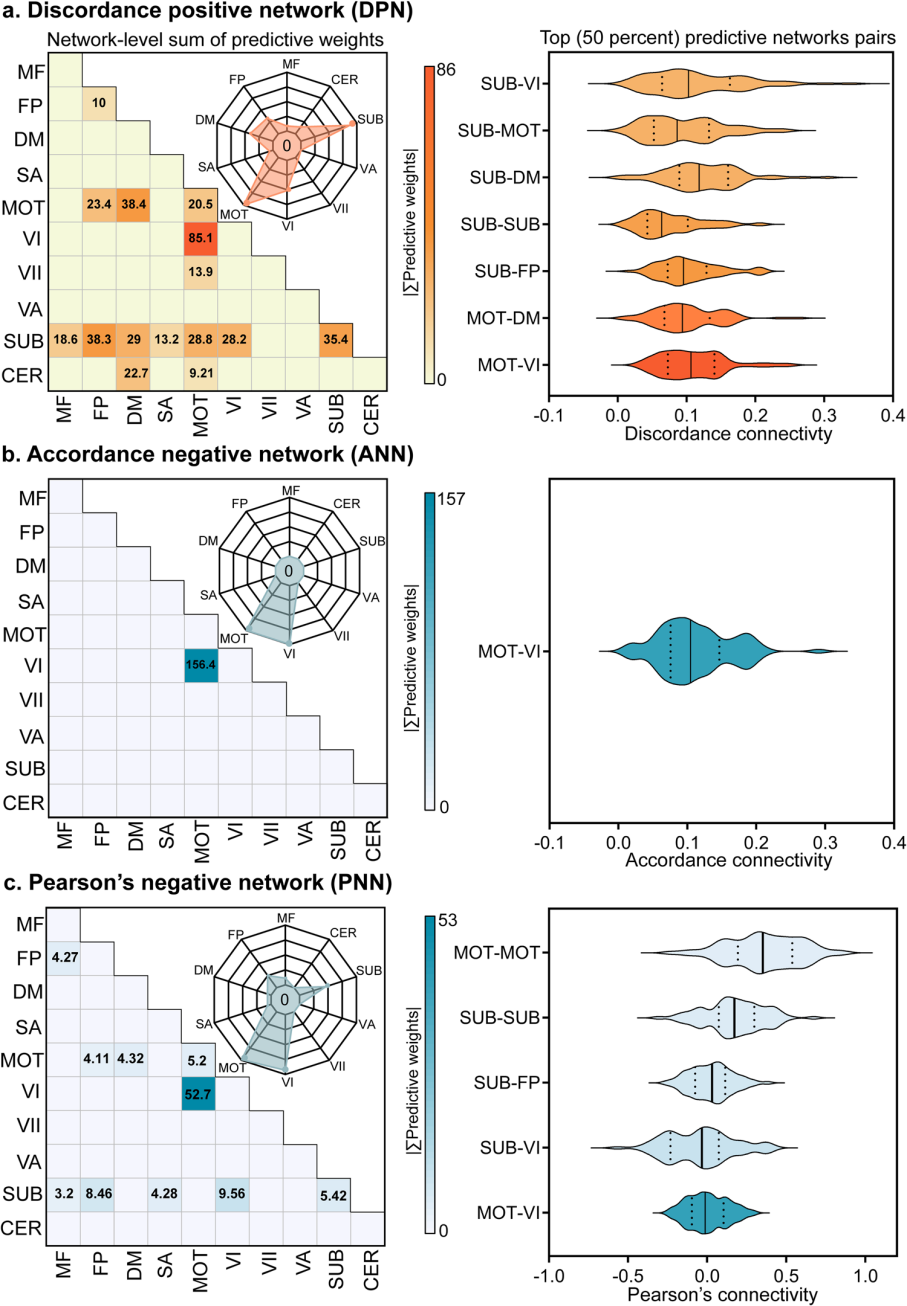
Network level representation of predictive weights and the distribution of top 50% network pairs on the discovery cohort (*n* = 71). 268 ROIs were divided into 10 canonical functional networks, and we characterized the whole predictive networks (**a**–**c**) from the edge-level (within- and between- networks, shown as heatmaps) and node-level (shown as radar charts) by aggregating all related weights. The first half of the predictive network pairs were selected according to the sorted weights, and the mean strength of network interactions were shown in the second column. The black solid lines in the violin plots represents for the median value, and the dash lines represents for the upper and lower quartiles in each network pair. Networks acronyms: MF Medial Frontal, FP Frontal Parietal, DM Default Mode, MOT Motor, VI Visual I, VII Visual II, VA Visual Association, SUB Subcortical, CER Cerebellum.

Disclosing the main drivers in predictive models is important to understand how the neurobiological substrates underpinned the motor function in PD. Hence, we assigned 268 nodes to 10 canonical functional networks according to previous literature^20,23,35^ to visualize predictive weights on the network level (Fig. 3). We first quantified the predictive weights belonging to each intra- or inter-network pair by summing up the regression coefficients of every connectivity (the heatmaps in Fig. 3). Thereafter, we sketched the distributions of the top fifty percent network pairs (top seven pairs for DPN, top one for ANN, and top five for PNN) by averaging the connected strength between nodes on the discovery cohort (the right column in Fig. 3). The most reliable negative or antagonistic (that is, higher UPDRS III motor scores with more anti-coupling) predictive weights across three candidate models were found in the pathway between motor network (MOT) and primary visual network (VI). In addition, the DPN model revealed that MOT, subcortical (SUB), default mode (DM), and frontoparietal (FP) had notable predictive weights, which also covered the results of ANN and PNN (see the radar plots in Fig. 3). These finding indicated that the motor function of PD might be represented as distributed decoupling of multi-level networks responsible for sensory perception, action selection, sequencing and planning^12,36^, and executive control processes^37^.

### External validations

**Fig. 4:**
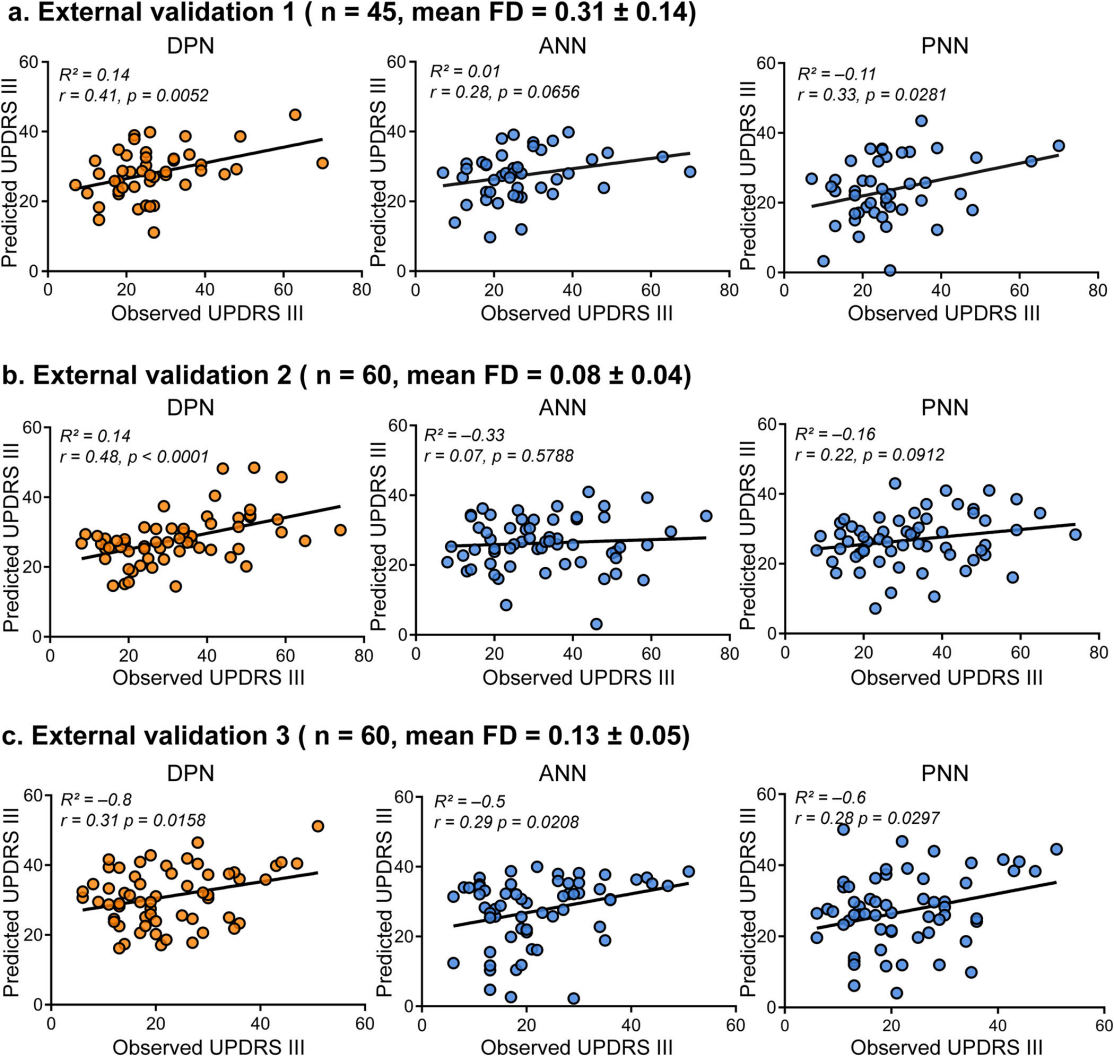
External validations of three candidate models. We showed the sensitivity and generalizability on three independent datasets. **a** External validation 1 (Study 2, *n* = 45) is based on a sample of the same center as Study 1, and participants have higher head movements (mean FD > 0.2 mm, translation > 3 mm, or rotation > 3°). **b** External validation 2 (Study 3, *n* = 60) is based on another center with a different scanner, and participants were recruited under the same head motion inclusion criteria as Study 1. **c** External validation 3 (Study 4, *n* = 60) was performed on the cohort from PPMI, which covered seven sites and the white race. The plots showed the relationships between the observed scores versus predicted scores. Each dot represents an individual participant, and the line represents the regression line. Combining all external validations, DPN showed better generalizability than the other two models. PPMI Parkinson’s Progression Markers Initiative.

For the blueprint of clinical translation, validating the generalizability of predictive models is crucial. Here, three independent and heterogeneous PD samples (Study 2 to Study 4) served as external validations. The heterogeneity between these test samples and the discovery cohort in Study 1 was demonstrated in Table 1. Study 2 (n_1_ = 45) included patients with PD belonging to the same center (TMMU) as Study 1, which possessed more severe head movements and never appeared in Study 1. As shown in Fig. 4a, the DPN model outperformed the other two in generalizability within this cohort (DPN: *R*^2^ = 0.14, *r* = 0.41, *P* = 0.0052; ANN: *R*^2^ = 0.01, *r* = 0.28, *P* = 0.0656; PNN: *R*^2^ = − 0.11, *r* = 0.33, *P* = 0.0281). Study 3 (n_2_ = 60) was recruited from a different center (CSU) with the same inclusion criteria in Study 1. As shown in Fig. 4b, this result also demonstrated that the DPN had robust predictive performance (DPN: *R*^2^ = 0.14, *r* = 0.48, *P* < 0.0001), whereas the other two models could hardly predict the observed UPDRS III scores (ANN: *R*^2^ = − 0.33 *r* = 0.07, *P* = 0.5788; PNN: *R*^2^ = − 0.16, *r* = 0.22, *P* = 0.0912). Study 4 used the most heterogeneous sample (n_3_ = 60), which consisted of seven different sites and other ethnicities (the white race accounted for 93%). Although the R-squared was negative in all predictive models, the *r* values were positive between the observations and predictions as shown in Fig. 4c (DPN: *R*^2^ = − 0.8, *r* = 0.31, *P* = 0.0158; ANN: *R*^2^ = − 0.5, *r* = 0.29, *P* = 0.0208; PNN: *R*^2^ = − 0.6, *r* = 0.28, *P* = 0.0297). After all validations, DPN showed better sensitivity and generalizability than the other two models (ANN and PNN), and it was defined as the final predictive model in this article.

**Table 1:** Demographic and clinical characteristics of all samples.

Variables	Study 1	Study 2	Study 3	Study 4	*P* value		
	(*n* = 71)	(*n* = 45)	(*n* = 60)	(*n* = 60)	1 vs 2	1 vs 3	1 vs 4
Age, years	60.04 ± 10.60 (28–83)	64.47 ± 9.41 (45–81)	53.58 ± 10.93 (24–69)	60.67 ± 7.88 (48–75)	0.021	<0.001*	0.699
Sex, male/female	29/42	30/15	33/27	41/19	0.012*	0.150	0.003*
Duration, years	5.92 ± 5.33 (0.17–32)	6.24 ± 4.61 (0.67–23)	7.13 ± 4.96 (0–20)	0.58 ± 0.68 (0.03–2.66)	0.733	0.180	<0.001*
Education, years	8.75 ± 3.83 (0–16)	8.03 ± 4.52 (0–18)	9.47 ± 3.61 (0–16)	15.17 ± 2.99 (8–22)	0.378	0.273	<0.001*
UPDRS III	26.30 ± 14.27 (2–67)	27.04 ± 12.81 (7–70)	33.45 ± 16.47 (8–73)	22.22 ± 10.63 (6–51)	0.770	0.009*	0.064
H&Y stage	2.15 ± 0.74 (1–5)	2.41 ± 0.71 (1–4)	2.51 ± 0.94 (1–5)	1.73 ± 0.49 (1–3)	0.066	0.020	<0.001*
MoCA^#^	21.82 ± 5.33 (5–30)	20.87 ± 5.03 (10–29)	22.26 ± 5.54 (6–30)	26.88 ± 2.81 (15–30)	0.335	0.652	<0.001*
Mean FD	0.11 ± 0.04 (0.03–0.19)	0.31 ± 0.14 (0.10–0.66)	0.08 ± 0.04 (0.02–0.18)	0.13 ± 0.05 (0.05–0.25)	<0.001*	<0.001*	0.007*
LEDD^#^	673.83 ± 236.76 (225–1350)	690.71 ± 148.52 (332–1089)	404.08 ± 214.37 (0–947.63)	355.49 ± 189.85 (50–800)	0.638	<0.001*	<0.001*

Notes: Values of variables are presented as mean ± SD (range). Statistical differences are listed between Study 1 and Study 2 (1 vs 2), Study 1 and Study 3 (1 vs 3), and Study 1 and Study 4 (1 vs 4). Chi-square test was performed on the sex, and two-sample *t*-test was performed on other variables. * indicates for the Bonferroni-corrected *P* < 0.05. ^#^ indicates for partially missed scores (MoCA: for Study 3 *n* = 7; LEED: for Study 4 *n* = 21). UPDRS III Unified Parkinson’s Disease Rating Scale motor subsection, SD Standard deviation, FD Framewise displacement, H&Y Hoehn and Yahr, MoCA Montreal Cognitive Assessment, LEDD levodopa equivalent daily dose.

It is also important to test whether the DPN-based model has the specificity to predict motor function in PD. Based on correlation analysis, we found no significant *r* values between the predicted UPDRS III scores and the variables of age (Study 1: *r* = 0.001, *P* = 0.992; Study 2: *r* < 0.001, *P* = 0.9998; Study 3: *r* = −0.082, *P* = 0.5344; Study 4: *r* = −0.017, *P* = 0.8974), mean framewise displacement (FD) (Study 1: *r* = −0.077, *P* = 0.5185; Study 2: *r* = −0.042, *P* = 0.7816; Study 3: *r* = −0.008, *P* = 0.9492; Study 4: *r* = −0.013, *P* = 0.9236), education (Study 1: *r* = –0.224, *P* = 0.0607; Study 2: *r* = −0.115, *P* = 0.4508; Study 3: *r* = −0.119, *P* = 0.3706; Study 4: *r* = −0.099, *P* = 0.4506), or Montreal Cognitive Assessment (MoCA) scores (Study 1: *r* = −0.207, *P* = 0.0838; Study 2: *r* = –0.052, *P* = 0.7338; Study 3: *r* = −0.215, *P* = 0.1224; Study 4: *r* = −0.223, *P* = 0.0872). We also found that the predicted scores had no significant differences between males and females by two-sample t-test (Study 1: *t* = −0.909, *P* = 0.367; Study 2: *t* = 0.802, *P* = 0.4299; Study 3: *t* = −1.133, *P* = 0.2625; Study 4: *t* = −0.580, *P* = 0.565). These results provided evidence for the specificity of the final model to predict motor function in PD (shown in Supplementary Fig. 6).

### Stage-specific effects of the DPN predictive model

**Fig. 5:**
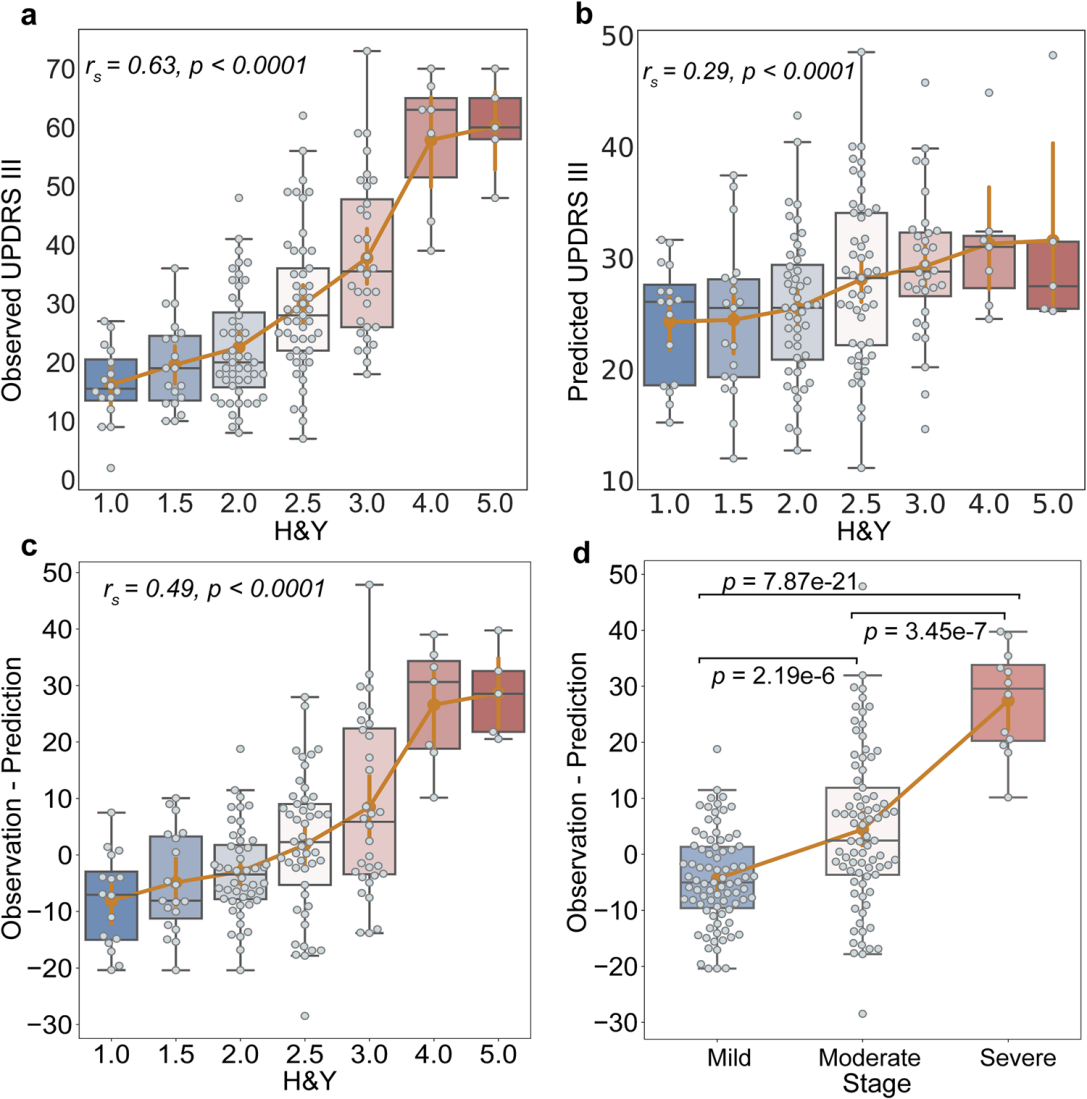
Stage-specific analysis of the DPN predictive model. Using the samples showed a relatively accurate performance (*R*^2^ > 0), Study 1 to 3 (*n* = 176) were aggregated to evaluate the predictive performance of DPN along with PD progression. **a** We delineated the observed UPDRS III scores at different H&Y stages, and found a significant correlation (Spearman’s *r* = 0.63, *P* < 0.0001). **b** We showed the relationship between predicted UPDRS III scores and H&Y stages (Spearman’s *r* = 0.29, *P* < 0.0001). **c** We correlated the residuals between observations and predictions with H&Y stages (Spearman’s *r* = 0.49, *P* < 0.0001). **d** Based on the H&Y, patients were divided into mild (H&Y = 1, 1.5, 2; *n* = 83), moderate (H&Y = 2.5, 3; *n* = 81), and severe (H&Y = 4, 5; *n* = 12) groups. Significant differences in predicted deviations were revealed between each pair of groups using two-sample *t*-tests. Boxplots indicate the median (the black line in the box), upper and lower quartiles (box), 1.5 times interquartile range (whiskers) and outliers (circles) for the values in each stage. DPN discordance predictive model.

Although the DPN model showed the best sensitivity to clinical motor function in PD and a good generalizability on different samples, a fine-grained portrait of predictive performance was further needed as the neuroimaging signature may have different patterns with the progression of PD^17^. Here, focusing on the samples with relatively accurate predicted outcomes (*R*^2^ > 0, Study 1 to Study 3, *n* = 176), we evaluated the consistency of the predictive performance on different stages based on Hoehn and Yahr (H&Y). We first found a significant correlation between the observed UPDRS III scores and H&Y stages (*r*_s_ = 0.63, *P* < 0.0001, Fig. 5a). Although predicted UPDRS III scores were also correlated with H&Y stages (*r*_s_ = 0.29, *P* < 0.0001, Fig. 5b), the slope of ascent was slower in the later stages. Thus, we correlated the residuals between observations and predictions with H&Y stages (*r*_s_ = 0.49, *P* < 0.0001, Fig. 5c) to investigate how the predictive performance covaried with disease degeneration in this study. Furthermore, we divided all patients into the three groups based on H&Y stages (Fig. 5d). Using one sample t test on the residuals, the mild group showed a significant tendency to predict higher scores (*t* = −4.76, *P* < 0.0001). Significant differences between each pair of subgroups were also found by using two-sample *t* test (*P* < 0.0001 Bonferroni corrected, Fig. 5d).

### Motor signature derived from the discordance predictive model

**Fig. 6:**
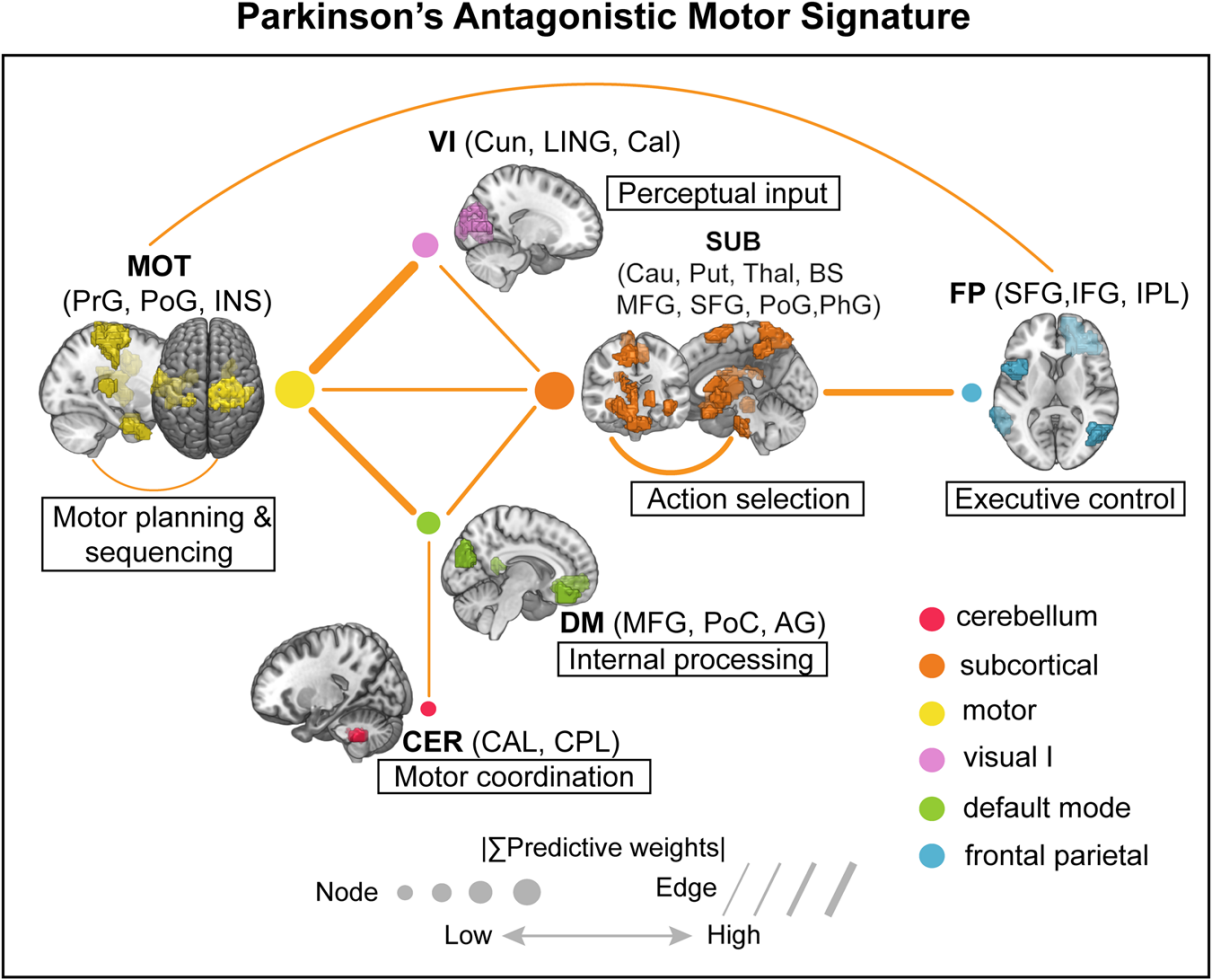
Parkinson’s antagonistic motor signature (PAMS): a functional connectivity biomarker of motor dysfunction in PD. We sketched the top ten predictive network pairs in the final model. The widths of dots and lines are proportional to the predictive weights. We also showed the function of each network according to previous literature and the corresponding anatomical regions. PrG precentral gyrus, PoG postcentral gyrus, INS insula gyrus, Cun cuneus, LING lingual gyrus, Cal calcarine, Cau caudate; Put putamen, Thal thalamus; BS brainstem, MFG medial frontal gyrus, SFG superior frontal gyrus, PhG parahippocampa gyrus; IFG inferior frontal gyrus, IPL inferior parietal lobe, PoC posterior cingulate, AG angular gyrus, CAL cerebellum anterior lobe, CPL cerebellum posterior lobe.

We further provided detailed anatomical information and interpretability about the final predictive model of motor function in PD. We dubbed Parkinson’s antagonistic motor signature (PAMS) for ease of further references and validations and summarized the top 10 network interactions from Fig. 3a. The size of nodes and lines in Fig. 6 was associated with the predictive weights, and more detailed information about the primary nodes (anatomical areas, predictive weights, etc.) and edges (regression coefficients) is listed in Supplementary Tables 2 and 3. Overall, the PAMS was characterized by functional decoupling among the MOT, SUB, FP, DM, and CER networks. Notably, MOT and SUB networks showed the almost equal dominance in PAMS, and the predictive weight on MOT-VI antagonistic interaction ranked first in all network-pairs. Moreover, we excluded the influence on the functional network strength of PAMS from the gray matter (GM) volumes (Study 1: *r* = 0.066, *P* = 0.5832; Study 2: *r* = 0.058, *P* = 0.7065; Study 3: *r* = −0.168, *P* = 0.1981; Study 4: *r* = 0.222, *P* = 0.0883; Supplementary Fig. 7a), and white matter (WM) volumes (Study 1: *r* = −0.046, *P* = 0.7032; Study 2: *r* = 0.071, *P* = 0.6413; Study 3: *r* = −0.091, *P* = 0.4916; Study 4: *r* = 0.060, *P* = 0.6482; Supplementary Fig. 7b).

## Discussion

In this study, we developed an antagonistic model characterized by discordance to form the mapping between brain systems and motor function in PD. Comparisons tests of candidate predictive models approved that the antagonistic profile of FC had the best sensitivity to reflect the motor dysfunction in PD (Study 1). Moreover, the antagonistic model showed good generalizability and robustness across heterogenous PD fMRI datasets (Study 2 to Study 4). We dubbed the model as PAMS and revealed that the PAMS was dominated by antagonistic interactions among the SUB, MOT, VI, CER as well as DM and FP networks. Notably the MOT-VI pathway accounted the most part of predictive weights among network-pairs and was consistent in all candidate models with different profiles of FC. Additional stage-specific analysis showed that the predicted scores generated from the PAMS achieved the best accuracy and tended to be higher than observed scores in the early course of PD, which indicated that the brain signature may be more prone to vary with the neurodegeneration process than clinical behaviors. Collectively, our results suggest that the clinical motor dysfunction of PD can be reflected by the multi-level antagonistic network interactions and provide a potential neuroimaging biomarker to evaluate motor function of PD patients in the early course.

Assuming the multiplex interactions within the human connectome, we adopted other profiles of FC to decompose the traditional Pearson’s connectivity into coupled interactions and anti-coupled interactions. We demonstrated that the final model featured by discordance showed the best sensitivity and generalizability to predict motor function in PD. It has been suggested that models would be more predictive if the assumed features fit the underlying nature of the brain representations involved^38^. Therefore, antagonistic interactions seem to be closely related to the neurobiological process of PD, which may be caused by dopamine deficiency. Similar observations were also found in the context of predicting long-term memory scores of mild cognitive impairment patients (another neurodegenerative disease)^39^. These findings are consistent with the “network degeneration hypothesis” supported by Seeley et al.^40^ suggesting that neurodegenerative diseases can selectively damage the highly coupled patterns of brain functional networks.

The antagonistic model showed that highly distributed brain networks participated in the motor dysfunction, which buttressed the opinion of Filippi et al.^17^ that not only sensorimotor circuitry but also non-motor networks evolved with the progression of PD. We found that between-network interactions accounted for 85.3% of the whole predictive weights (Fig. 3), suggesting that normal motor function primarily relied on the coordinated activities among different brain networks. With a similar perspective as Rosenberg et al.^41^, we considered motor ability as an emergent property of distributed networks. We found that the MOT and SUB networks appeared as the most important predictors in the final model, which accounted for 92.7% of the whole predictive weights. This observation keeps in line with the well-known hallmark of PD regarding the reduced dopamine input to cortices (particularly the sensorimotor network) and subcortical regions^12^. Notably, the interaction between MOT and VI was the most important predictor among network-pairs (accounted for 18.9% of the weights, Fig. 3). The dorsal “action” stream, which is known as the “where” pathway, also plays an important and direct role in motor planning by integrating perceptual input (such as visual spatial information) into somatomotor areas. Thus, MOT-VI antagonism may reflect the perceptual decoupling between perceptual system and motor-planning system (dampened integration of visual input), which are the characteristics of patients with PD^42,43^. This result could also provide a neurological explanation for the fact that gait performance could be improved by combining external visual cues^44,45^. Furthermore, the importance of MOT-VI suggested that new therapies aiming to improve the coordination between visual and motor systems may have great potential in enhancing the kinetic performance of PD patients.

Besides the consistent pattern with previous literature^36^, our model also opened a new window onto the role of high-order brain networks (DM and FP) in motor dysfunction. Numerically, the MOT-DM and SUB-FP accounted for 8.54% and 8.52% of the predictive weights respectively. The functional decoupling between the frontoparietal cortices (responsible for execution and control) and subcortical structures may be a mechanism responsible for impaired efficiency of motor control^12,37,46^. Notably, the DM, which is traditionally considered as an internal processor related to self-referential processing^47^, was emphasized in the final model. From a topological perspective, the DM seemed to be a hub port among MOT, SUB, and CER (Fig. 6), reflecting the participation of DM into the cardinal motor system. This finding could be supported by recent neuroimaging studies^48,49^ that revealed decreased functional interactions of cognitive networks (including the DM) in cognitively unimpaired PD patients with akinetic/rigid symptoms. The decreased connectivity within sensorimotor and default mode networks was also related to the freezing of gait^29^. This result suggested that the role of DM might be overlooked in the process of motor dysfunction in PD and more attention should be paid into the internal processor in the future research (e.g. whether the mediation exercise could have a positive effect on motor function).

As the degenerative nature of PD, clarifying the predictive performance on different stages is crucial for clinical translation. We found that the prediction residuals of the final predictive model were significantly associated with the H&Y values (Fig. 5c). Significant differences were also observed among the three groups (Fig. 5d). Notably, the significant difference between the mild group (H&Y = 1, 1.5, 2) and moderate group (H&Y = 2.5, 3) might correspond to the pivotal milestone in the development of PD (the transition from H&Y stage II to III)^50^. Prior research suggested that nearly 60% of substantia nigra pars compacta dopaminergic neurons had already been lost at the onset of PD clinical symptoms^51^, indicating that the actual damage of neuron systems could be more severe than the behavioral observations in this course. Thus, we infer that the observations of significantly higher motor scores generated from brain signature could be partially caused by the lagging effect between the observed clinical motor symptoms and severe dopaminergic loss in PD patients of mild group. It seems that there tends to be a time delay for PD patients in the early stages to manifest the actually damaged degree of neural systems at the behavioral level. Our stage-specific analysis provides novel insights into the brain antagonistic signature PD and suggests that the final predictive model could be set as an early alarm for PD patients.

The present study is limited in several ways, but it lays the groundwork for additional future investigations. First, the final predictive model was trained on the Chinese population (Asian race). Although we tested the model on other ethnicity (the white race) collected from the PPMI, the generalizability was not as good as that of Study 2 and 3. As previous literature has demonstrated that genetic and environmental factors could contribute to the heterogeneity of PD^52^, the predictive models based on different ethnicities are further needed. Second, different motor symptoms rely on not only overlapping brain regions but also distinctive patterns of neural activity in PD^36,53^. Thus, future phenotype-specific predictive models would lead to more fine-grained delineation of Parkinson’s motor neural signature and provide more values in clinical applications. Third, the current study focuses on the functional signature of motor function of PD, although we have demonstrated that the motor-related functional signature had insignificant sensitivity to predict cognitive function in PD, it is also important to explore the cognition-specific signature and clarify the potential shared and distinct patterns with motor-specific signature (e.g. with dementia or without dementia) in the future work. Additionally, our current results on antagonistic functional connectivity and motor function of PD likely reflect a mixture of direct and indirect connections between brain regions. Future studies may combine both structural modality (e.g. diffusion tensor imaging) and fMRI in order to assess the joint contribution of structural and functional connectivity to motor dysfunction in PD patients. Finally, fewer samples in late stages (only 12 patients in the severe group) could also affect the generalizability of the final predictive model. Future studies that recruit more patients in the late stages could fill the vacancy.

In summary, the current study utilized antagonistic interactions within the functional connectome to identify a reliable Parkinson’s motor signature via CPM framework. We demonstrated that PAMS showed high sensitivity and generalizability across four independent PD cohorts. Motor dysfunction of PD could be represented as distributed antagonistic systems, particularly in SUB, MOT, VI, CER as well as DM and FP networks. In the context of clinical translation, the final predictive model has a great potential to evaluate motor function of PD at the early stages and to develop new behavioral therapies for PD.

## Methods

### Clinical assessments

Study 1 to 3 were approved by the ethics committee of the TMMU (Chongqing, China) and CSU (Changsha, China). All participants (n = 176) provided written informed consents in line with the Declaration of Helsinki (DoH), and they were diagnosed with PD according to the UK Parkinson’s Disease Society Brain Bank criteria^54^ by at least two or more experienced neurologists. Patients were scanned under the off-medication state, at least 12 h after using any antiparkinsonian drug. Only the patients without any other neurological or psychiatric disorders (such as seizures, stroke, severe depression, or claustrophobia) were recruited in this study. Study 4 from PPMI was conducted in accordance with the DoH and the Good Clinical Practice guidelines after approval of the local ethics committees of the participating sites^31^, including Baylor College of Medicine, Emory University, Tulane University et al. Participants in Study 4 (n = 60) also provided written informed consents.

Patients’ motor function was clinically assessed by the part III of the UPDRS^55^ and the H&Y scale^56^. Cognitive function was assessed by the MoCA scale^57^. Levodopa equivalent daily dose (LEDD)^58^ was also recorded for PD patients with the online Levodopa Equivalent Dose Calculator (https://www.parkinsonsmeasurement.org/toolBox/levodopaEquivalentDose.htm). All participants were assessed before scanning under an off-medication state. Notably, clinical motor scores in Study 4 are assessed by the Movement Disorder Society-sponsored revision of the Unified Parkinson’s Disease Rating Scale (MDS-UPDRS) part III, which consists of 18 items compared with the original scale (14 items)^59^. All clinical assessments were showed in Table 1.

### MRI acquisition

From Study 1 to Study 3, subjects were instructed to close their eyes and stay awake during scanning. Meanwhile, subjects must keep their mind wandering without thinking of anything willfully. Foam padding and earplugs were used to alleviate head motion and scanner noise. Study 1 and Study 2 were collected from the same center (TMMU), and Study 3 belonged to another center (CSU) with a different scanner. For Study 4, we aimed to recruit patients scanned with the same equipment and parameters from PPMI. Besides, the scanning parameters in Study 4 were similar to those in Study 1. Detailed information was shown as below and was summarized in Table 2.

**Table 2:** Scanners and scanning parameters for each center.

	Study 1 and 2	Study 3	Study 4
Scanner	Siemens 3 T	GE 3 T	Siemens 3 T
Model name	Trio Tim	Signa HDxt	Trio Tim
Anatomical scan			
Weighting	T1	T1	T1
Sequence	MPRAGE	3D BRAVO	MPRAGE
TR (ms)	1900	7.79	2300
TE (ms)	2.52	2.98	2.98
IT (ms)	900	800	900
Resolution (mm^3^)	1×1×1	1×1×1	1×1×1
FOV (mm^2^)	256×256	256×256	240×256
Flip angle	9	7	9
Resting state fMRI			
Weighting	T2^*^	T2^*^	T2^*^
Sequence	EPI	EPI	EPI
TR (ms)	2000	2000	2400
TE (ms)	30	30	25
Resolution (mm^3^)	3.0×3.0×3.99	3.44×3.44×4.60	3.29×3.29×3.30
FOV (mm^2^)	192×192	220×220	224×217
Flip angle	90	90	80
Num. of slices	36	32	40
Slice thickness (mm)	3	4	3.3
Num. of vols	240	180	210
Scanning time	8 min	6 min	8 min 24 sec

TMMU: On a 3.0 T Siemens Trio Total imaging matrix (Tim) whole-body MRI system (Siemens Medical Solutions, Erlangen, Germany), functional data were collected transversely by using an echo-planar imaging (EPI) sequence with the following setting: TR = 2000 ms, TE = 30 ms, flip angle = 90°, FOV = 192 mm×192 mm, slice thickness = 3 mm, voxel size = 3.0 mm×3.0 mm×3.99 mm, and 36 slices. For each subject, a total of 240 volumes were obtained from the scan time of 8 min. Structural 3D T1-weighted images were acquired using a magnetization-prepared rapid gradient-echo (MP-RAGE) sequence for co-registration with functional images: TR = 1900 ms, TE = 2.52 ms, flip angle = 9°, slice thickness = 1 mm, slices = 176, FOV = 256 mm×256 mm, matrix size = 256×256, and voxel size = 1 mm×1 mm ×1 mm.

CSU: All subjects were scanned on a 3.0 T GE Signa MR system (General Electric, Fairfield, CT, USA). Functional data were acquired using a gradient echo EPI sequence with the following parameters: TR = 2000 ms, TE = 30 ms, flip angle = 90°, FOV = 220 mm×220 mm, slice thickness = 4.0 mm, voxel size = 3.44 mm×3.44 mm×4.60 mm, and 32 slices. Finally, 180 volumes were acquired for each subject over 6 min. T1 images were also collected for normalization: TR = 7.792 ms, TE = 2.984 ms, flip angle = 7°, slice thickness = 1 mm, slices = 188, matrix size = 256×256, and voxel size = 1 mm×1 mm ×1 mm.

PPMI: All patients were scanned on a 3.0 T Siemens MRI system using the same sequence as TMMU and the parameters for functional images are as follows: TR = 2400 ms, TE = 25 ms, flip angle = 80°, FOV = 224 mm×217 mm, slice thickness = 3.3 mm, voxel size = 3.29 mm×3.29 mm×3.30 mm, and 40 slices. A total of 210 images were acquired for each participant in 8 min. The scanning parameters for T1 images are as follows: TR = 2300 ms, TE = 2.98 ms, flip angle = 9°, slice thickness = 1 mm, slices = 176, FOV = 240 mm×256 mm, matrix size = 240×256, and voxel size = 1 mm×1 mm ×1 mm.

### Image preprocessing

All data were preprocessed using Data Processing Assistant for Resting-State fMRI (DPARSF v5.0, http://www.restfmri.net)^60^, which was based on Statistical Parametric Mapping (SPM12, http://www.fil.ion.ucl.ac.uk/spm) under MATLAB 9.5 environment (https://www.mathworks.com). Before the preprocessing workflow, functional and structural scans were manually realigned along the anterior–posterior commissure (AC-PC) line. The first 10 volumes of functional scans were discarded to stabilize the magnetization of MRI signals, and then were corrected for the acquisition delay between slices and head movement. Structural images were co-registered to the remaining images and segmented into GM, WM, and CSF using DARTEL^61^. The resulting deformation field maps were retained for further spatial normalization of functional images. Regression Covariates included the signals from WM and CSF, Friston-24 head motion parameters (six motion parameters, six temporal derivatives, and their corresponding squares), and the whole brain were regressed in the native image space. Although global signal regression (GSR) is a controversial step, previous studies have indicated that GSR can not only remove physiological noise generated by head motion, particularly on movement disorders such as PD^62–64^, but also increase the dependency between FC and behavior^65^. Linear, quadratic, and cubic drifts were also removed from the time series. After normalization to Montreal Neurological Institute space and resampling to 3 mm×3 mm×3 mm, residual images were temporally smoothed with a band-pass filter (0.01–0.1 Hz) to retain the most neurological-related signals. According to head movements, excluded criteria of Study 1 were head translation > 3 mm, head rotation > 3°, and mean framewise displacement (FD) > 0.2 mm. Study 3 and Study 4 hold the same criteria as Study 1. Statistics of mean FD were shown in Table 1.

### Functional connectome construction

The nodes of the functional network were defined on the basis of the 268-node Shen brain atlas, which covered the whole brain regions, including cerebral cortex, subcortical, and cerebellum^66^. We extracted the average signal of each node to represent regional brain activity. In our present study, three profiles of FC were used to construct connectome: Pearson’s correlation coefficients, the most popular measure based on the method of covariance; and two recently developed measures (accordance and discordance) based on the temporal analysis of extreme points^28,39,67^.

To calculate accordance and discordance, we first normalized each BOLD signal by subtracting its own mean and dividing by the standard deviation. In finding extreme events, we set a predefined quantile of *q* = 0.9 as the threshold on continuous time courses, and each normalized time series *z* was then divided into two discrete binarized series: activation series *z*^*u*^ and deactivation series *z*^*l*^. We used the same definition of positive threshold vector *z*^*u*^ and negative threshold vector *z*^*l*^ as that described by Yoo et al.^67^

1 $$\begin{array}{*{20}{c}} {\forall \;timepoint\;t,z_t^u = 0\;if\;z_t \,<\, {\Phi}^{ - 1}\left( q \right),and\;z_t^u = 1\;otherwise} \end{array}$$

2 $$\begin{array}{*{20}{c}} {\forall \;timepoint\;t,z_t^l = 0\;if\;z_t \,>\, {\Phi}^{ - 1}\left( {1 - q} \right),and\;z_t^l = 1\;otherwise} \end{array}$$

where ${{{\mathrm{{\Phi}}}}}^{ - 1}$ is the inverse cumulative distribution function; $a_{ij}$ and $d_{ij}$ represent the accordance and discordance between each pair of nodes respectively, which were calculated by using the following equations:

3 $$\begin{array}{*{20}{c}} {a_{ij} = \left( {z_i^u \ast z_j^u + z_i^l \ast z_j^l} \right)/\sigma _i\sigma _j} \end{array}$$

4 $$\begin{array}{*{20}{c}} {d_{ij} = \left( {z_i^u \ast z_j^l + z_i^l \ast z_j^u} \right)/\sigma _i\sigma _j} \end{array}$$

where $$\sigma _i = \sqrt {z_i^u \ast z_i^u + z_i^l \ast z_i^l}$$

### Predictive model training and validations

In this study, we adopted the CPM framework to identify FC patterns underpinning motor dysfunction of PD patients. As shown in Fig. 1, connectivity matrices and UPDRS III scores served as inputs, followed by feature selection, model building, and model validation steps. The trained models were applied to unseen individuals to test their sensitivity and generalizability. Notably, head movements can contribute to a main and complex confounding effect in CPM analysis, as robust but spurious patterns of connectivity can be introduced by large amounts of head motion^22^. Therefore, before CPM analysis, we first tested the dependency between mean FD and UPDRS III scores using Pearson’s correlation. For feature selection, given the reported dependency between aging and FC^68^, predictive features were defined by partial correlation method. That is, when we correlated FC with motor scores, age was set as a confounder. If an edge was positively associated with UPDRS III scores (*r* > 0, *P* < 0.001), this edge would be assigned to a positive network, and if an edge had a negative correlation (*r* < 0, *P* < 0.001) with scores, this edge would be assigned to a negative network. In model building, partial least square regression (PLSR) was adopted to learn the regression line and provide predictive weights on each feature, which can eliminate multicollinearity in predictor variables by representing inputs with fewer components (here we used the first component). Then the predictive performance was evaluated with a LOOCV method on the discovery cohort (Study 1, n = 71), which reduced the risk of overfitting by removing one subject from training data and building a predictive model on N–1 subjects. After obtaining predicted scores on all subjects, we used two estimators to quantify the sensitivity of models. One is Pearson’s correlation between predicted motor scores and observed motor scores, and the other is predicted R-squared (*R*^*2*^). The latter estimator represented the explained variance between predicted values and observed values, as defined by the following equation:

5 $$\begin{array}{*{20}{c}} {R^2 = 1 - \frac{{SSE}}{{SST}}} \end{array}$$

where *SSE* is the sum of squared error, and *SST* is the sum of squared total. Notably, a negative correlation indicated an unsuccessful prediction. We could define candidate models with fixed parameters on the whole discovery set for further validations and applications.

As the nature of cross-validation, the predictive features could be slightly different in each iteration. We finally defined the predictive networks by retaining the edges that showed a significant correlation (*P* < 0.001) at least 95% of the iterations. More stringent threshold (100%) and looser threshold (90%) were also used for visualization and comparison. For assessing the generalizability, the candidate predictive models were directly applied to other independent datasets (Study 2, *n* = 45; Study 3, *n* = 60; Study 4, *n* = 60) for external validations. Combining all validations in this work, we could find the final predictive model with the best sensitivity and generalizability. In addition, we further tested the relationships between the predicted UPDRS III scores and extraneous variables (age, mean FD, sex, education, and cognition) in a post hoc analysis to validate the specificity of the motor-related brain signature. We also investigated the covariance between the structural properties of brain and the PAMS strength. Specifically, the absolute volumes of GM and WM were first estimated using the Computational Anatomy Toolbox (CAT12, http://www.neuro.uni-jena.de/cat/). Thereafter the volumes were associated with the network strength of PAMS (the sum of antagonistic interactions) by Pearson’s correlation.

### Statistical analysis

In the internal validation, we first obtained the parametric *P* value of the correlation coefficient between predicted scores and observed scores. However, considering that the number of degrees of freedom in LOOCV was overestimated^23^, a permutation test was adopted. We first shuffled the UPDRS III scores and then performed the same predictive method. By randomly permuting the motor scores 10,000 times, a null distribution of correlation values between observations and predictions was obtained. *P*_permutation_ was calculated as the ratio of the number of values from the null distribution that was larger or equal to the true correlation to the number of permutations.

By using three profiles of FC, we obtained different predictive models to map brain systems to behaviors. To determine which model is the best, Steiger’s z test^69^ was used to compare the difference between two dependent correlation coefficients (values between each pair of predicted scores and observed scores). Sterger’s z test was performed with the “cocor” package^70^ in R programming (R version 3.6.3).

### Stage-specific analysis of the final predictive model

Given the progression nature of PD, the mapping between functional connectome and individual behavior might vary across disease stages^17^. We further investigated how the predictive performance of the final model varied with H&Y stages to provide more precise illustrations for clinical translation. Notably, this stage-specific analysis only focused on the results with a positive R-squared value (Study 1, Study 2, and Study 3). We first correlated observed and predicted UPDRS III scores with H&Y stages and then correlated the prediction errors (residuals between predictions and observations) with stages. According to previous literature^71^, we divided all subjects into three subgroups from mild motor impairment to severe motor impairment (mild group: H&Y stages = 1, 1.5, 2.0; moderate group: H&Y stages = 2.5, 3.0; severe group: H&Y stages = 4.0, 5.0). Finally, we compared the prediction errors between each pair of subgroups using two sample *t*-test.

### Reporting summary

Further information on research design is available in the Nature Research Reporting Summary linked to this article.

## Supplementary information

supplemental tables and figures

Reporting Summary

## Data Availability

Data for the PPMI are freely available in the public domain through the Parkinson’s Progression Markers Initiative website (https://www.ppmi-info.org/) as detailed in the Methods section. Data for the TMMU and CSU are available from the corresponding author subject to anonymization to protect privacy of clinical data and implementation of a data sharing agreement as required by the local IRB.

## Code availability

All codes for defining the PAMS and supporting the main statistical analyses described in the Methods section are publicly available at Github (https://github.com/XuyangWang96/PAMS).

## References

[1] Willis AW (2013). Parkinson disease in the elderly adult. Mo. Med..

[2] Tessitore A, Cirillo M, De Micco R (2019). Functional connectivity signatures of Parkinson’s disease. J. Parkinsons Dis..

[3] McGregor MM, Nelson AB (2019). Circuit mechanisms of Parkinson’s disease. Neuron.

[4] Tysnes OB, Storstein A (2017). Epidemiology of Parkinson's disease. J. Neural Transm.

[5] Palop JJ, Chin J, Mucke L (2006). A network dysfunction perspective on neurodegenerative diseases. Nature.

[6] Caligiore D (2016). Parkinson’s disease as a system-level disorder. npj Parkinson’s Dis..

[7] Bressler SL, Menon V (2010). Large-scale brain networks in cognition: emerging methods and principles. Trends Cogn. Sci..

[8] Woodward ND, Cascio CJ (2015). Resting-state functional connectivity in psychiatric disorders. JAMA Psychiatry.

[9] Mulders PC, van Eijndhoven PF, Schene AH, Beckmann CF, Tendolkar I (2015). Resting-state functional connectivity in major depressive disorder: a review. Neurosci. Biobehav. Rev..

[10] Hull JV (2016). Resting-state functional connectivity in autism spectrum disorders: a review. Front Psychiatry.

[11] Gao L-L, Wu T (2016). The study of brain functional connectivity in Parkinson’s disease. Transl. Neurodegeneration.

[12] Sharman M (2013). Parkinson’s disease patients show reduced cortical‐subcortical sensorimotor connectivity. Mov. Disord..

[13] Herz DM, Eickhoff SB, Løkkegaard A, Siebner HR (2014). Functional neuroimaging of motor control in Parkinson’s disease: A meta‐analysis. Hum. brain Mapp..

[14] Szewczyk-Krolikowski K (2014). Functional connectivity in the basal ganglia network differentiates PD patients from controls. Neurology.

[15] Campbell MC (2015). CSF proteins and resting-state functional connectivity in Parkinson disease. Neurology.

[16] Postuma RB (2016). Resting state MRI: a new marker of prodromal neurodegeneration?. Brain.

[17] Filippi, M. et al. Longitudinal brain connectivity changes and clinical evolution in Parkinson’s disease. *Mol. Psychiatry* **26**, 5429–5440 (2021). 10.1038/s41380-020-0770-0 32409731

[18] Bzdok D, Ioannidis JPA (2019). Exploration, inference, and prediction in neuroscience and biomedicine. Trends Neurosci..

[19] Sui J, Jiang R, Bustillo J, Calhoun V (2020). Neuroimaging-based individualized prediction of cognition and behavior for mental disorders and health: methods and promises. Biol. Psychiatry.

[20] Finn ES (2015). Functional connectome fingerprinting: identifying individuals using patterns of brain connectivity. Nat. Neurosci..

[21] Gratton C (2018). Functional brain networks are dominated by stable group and individual factors, not cognitive or daily variation. Neuron.

[22] Shen X (2017). Using connectome-based predictive modeling to predict individual behavior from brain connectivity. Nat. Protoc..

[23] Rosenberg MD (2016). A neuromarker of sustained attention from whole-brain functional connectivity. Nat. Neurosci..

[24] Beaty RE (2018). Robust prediction of individual creative ability from brain functional connectivity. Proc. Natl Acad. Sci. USA.

[25] Yoo, K. et al. A brain-based universal measure of attention: predicting task-general and task-specific attention performance and their underlying neural mechanisms from task and resting state fMRI (Cold Spring Harbor Laboratory, 2021).

[26] Goldfarb EV, Rosenberg MD, Seo D, Constable RT, Sinha R (2020). Hippocampal seed connectome-based modeling predicts the feeling of stress. Nat. Commun..

[27] Lee JJ (2021). A neuroimaging biomarker for sustained experimental and clinical pain. Nat. Med.

[28] Meskaldji, D., Morgenthaler, S. & Ville, D. V. D. New measures of brain functional connectivity by temporal analysis of extreme events. *2015 IEEE 12th International Symposium on Biomedical Imaging (ISBI)*. 26–29 (2015).

[29] Canu E (2015). Brain structural and functional connectivity in Parkinson’s disease with freezing of gait. Hum. Brain Mapp..

[30] Filippi M, Sarasso E, Agosta F (2019). Resting state functional MRI in Parkinsonian syndromes. Mov. Disord. Clin. Pract..

[31] Marek K (2018). The Parkinson’s progression markers initiative (PPMI)–establishing a PD biomarker cohort. Ann. Clin. Transl. Neurol..

[32] Salehi M (2020). There is no single functional atlas even for a single individual: functional parcel definitions change with task. Neuroimage.

[33] Power JD (2011). Functional network organization of the human brain. Neuron.

[34] Fan L (2016). The Human Brainnetome atlas: a new brain atlas based on connectional architecture. Cereb. Cortex.

[35] Lichenstein SD, Scheinost D, Potenza MN, Carroll KM, Yip SW (2021). Dissociable neural substrates of opioid and cocaine use identified via connectome-based modelling. Mol. Psychiatry.

[36] Moustafa AA (2016). Motor symptoms in Parkinson’s disease: a unified framework. Neurosci. Biobehav. Rev..

[37] Shine JM (2013). Freezing of gait in Parkinson’s disease is associated with functional decoupling between the cognitive control network and the basal ganglia. Brain.

[38] Woo CW, Chang LJ, Lindquist MA, Wager TD (2017). Building better biomarkers: brain models in translational neuroimaging. Nat. Neurosci..

[39] Meskaldji DE (2016). Prediction of long-term memory scores in MCI based on resting-state fMRI. Neuroimage Clin..

[40] Seeley WW, Crawford RK, Zhou J, Miller BL, Greicius MD (2009). Neurodegenerative diseases target large-scale human brain networks. Neuron.

[41] Rosenberg MD, Finn ES, Scheinost D, Constable RT, Chun MM (2017). Characterizing attention with predictive network models. Trends Cogn. Sci..

[42] Uc E (2005). Visual dysfunction in Parkinson disease without dementia. Neurology.

[43] Davidsdottir S, Cronin-Golomb A, Lee A (2005). Visual and spatial symptoms in Parkinson’s disease. Vis. Res..

[44] Sidaway B, Anderson J, Danielson G, Martin L, Smith G (2006). Effects of long-term gait training using visual cues in an individual with Parkinson disease. Phys. Ther..

[45] Schlick C (2016). Visual cues combined with treadmill training to improve gait performance in Parkinson’s disease: a pilot randomized controlled trial. Clin. Rehabilitation.

[46] Tessitore A (2012). Resting-state brain connectivity in patients with Parkinson’s disease and freezing of gait. Parkinsonism Relat. Disord..

[47] Davey CG, Pujol J, Harrison BJ (2016). Mapping the self in the brain’s default mode network. Neuroimage.

[48] Karunanayaka PR (2016). Default mode network differences between rigidity- and tremor-predominant Parkinson’s disease. Cortex.

[49] Hou Y (2017). Default-mode network connectivity in cognitively unimpaired drug-naive patients with rigidity-dominant Parkinson’s disease. J. Neurol..

[50] Shulman LM (2008). The evolution of disability in Parkinson disease. Mov. Disord..

[51] Dauer W, Przedborski S (2003). Parkinson’s disease: mechanisms and models. Neuron.

[52] Ball N, Teo WP, Chandra S, Chapman J (2019). Parkinson’s disease and the environment. Front Neurol..

[53] Chen HM (2015). Different patterns of spontaneous brain activity between tremor‐dominant and postural instability/gait difficulty subtypes of Parkinson’s disease: a resting‐state fMRI study. CNS Neurosci. Therap..

[54] Hughes AJ, Daniel SE, Kilford L, Lees AJ (1992). Accuracy of clinical diagnosis of idiopathic Parkinson’s disease: a clinico-pathological study of 100 cases. J. Neurol. Neurosurg. Psychiatry.

[55] Movement Disorder Society Task Force on Rating Scales for Parkinson's Disease (2003). The unified Parkinson’s disease rating scale (UPDRS): status and recommendations. Mov. Disord..

[56] Hoehn MM, Yahr MD (1998). Pakinsonism: onset, progression, and mortality. Neurology.

[57] Dalrymple-Alford JC (2010). The MoCA: well-suited screen for cognitive impairment in Parkinson disease. Neurology.

[58] Tomlinson CL (2010). Systematic review of levodopa dose equivalency reporting in Parkinson’s disease. Mov. Disord..

[59] Goetz CG (2008). Movement disorder society-sponsored revision of the Unified Parkinson’s Disease Rating Scale (MDS-UPDRS): scale presentation and clinimetric testing results. Mov. Disord..

[60] Chao-Gan Y, Yu-Feng Z (2010). DPARSF: a MATLAB toolbox for “Pipeline” data analysis of resting-state fMRI. Front Syst. Neurosci..

[61] Ashburner J (2007). A fast diffeomorphic image registration algorithm. Neuroimage.

[62] Power JD, Laumann TO, Plitt M, Martin A, Petersen SE (2017). On global fMRI signals and simulations. Trends Cogn. Sci..

[63] Ciric R (2017). Benchmarking of participant-level confound regression strategies for the control of motion artifact in studies of functional connectivity. Neuroimage.

[64] Gratton C (2019). Emergent functional network effects in Parkinson disease. Cereb. Cortex.

[65] Li J (2019). Global signal regression strengthens association between resting-state functional connectivity and behavior. Neuroimage.

[66] Shen X, Tokoglu F, Papademetris X, Constable RT (2013). Groupwise whole-brain parcellation from resting-state fMRI data for network node identification. Neuroimage.

[67] Yoo K (2018). Connectome-based predictive modeling of attention: comparing different functional connectivity features and prediction methods across datasets. Neuroimage.

[68] Sala-Llonch R, Bartres-Faz D, Junque C (2015). Reorganization of brain networks in aging: a review of functional connectivity studies. Front. Psychol..

[69] Steiger JH (1980). Tests for comparing elements of a correlation matrix. Psychological Bull..

[70] Diedenhofen B, Musch J (2015). cocor: a comprehensive solution for the statistical comparison of correlations. PLoS ONE.

[71] Goetz CG (2004). Movement Disorder Society Task Force report on the Hoehn and Yahr staging scale: status and recommendations the Movement Disorder Society Task Force on rating scales for Parkinson’s disease. Mov. Disord..

