# Supplementary material for: Antagonistic network signature of motor function in Parkinson’s disease revealed by connectome-based predictive modeling
Source: NPJ Parkinsons Dis. 2022 Apr 22;8:49. doi: 10.1038/s41531-022-00315-w (PMC9033778; doi:10.1038/s41531-022-00315-w)
Supplement: Supplementary file 1 — supplemental tables and figures [file 41531_2022_315_MOESM1_ESM.pdf]

Supplementary Information for:

Antagonistic network signature of motor function in Parkinson's disease revealed by connectome-based predictive modeling

Xuyang Wang[1,#], Kwangsun Yoo[2,#], Huafu Chen[1*], Ting Zou[1], Hongyu Wang[1], Qing Gao[4], Li Meng[5*], Xiaofei Hu[3*], and Rong Li[1*]

[1]The Clinical Hospital of Chengdu Brain Science Institute, MOE Key Laboratory for Neuroinformation, High-Field Magnetic Resonance Brain Imaging Key Laboratory of Sichuan Province, School of Life Science and Technology, University of Electronic Science and Technology of China, Chengdu, 610054, P.R. China.

[2]Department of Psychology, Yale University, New Haven, CT 06520, USA

[3]Department of Radiology, Southwest Hospital, Third Military Medical University, Chongqing, 610054, P.R. China

[4]School of Mathematical Sciences, University of Electronic Science and Technology of China, Chengdu, 610054, P.R. China

[5]Department of Radiology, Xiangya Hospital, Central South University, Changsha, 410008, P.R. China

[#]These authors contributed equally to this work.

[*]Correspondence to: Rong Li, Email: rongli1120@gmail.com;

Huafu Chen, Email: chenhf@uestc.edu.cn;

Xiaofei Hu, Email: harryzonetmmu@163.com;

Li Meng, Email: mengli96130@csu.edu.cn;

Running title: Antagonistic motor signature of Parkinson's disease

Supplementary Tables:

Supplementary Table 1. Information about each edge in three network models

Brain labels	Lobes	Broadman area	MNI coordinates
**Discordance positive network (DPN)**			
(1) 1 - 197	Prefrontal_R – Temporal_L	BA11 – BA22	(13.9,56.8,-16.6) – (-57,-14.5,-6.9)
(2) 2 - 180	Prefrontal_R – Parietal_L	BA11 – BA40	(9.6,17.8,-19.5) – (-42.2,-31.2,15.9)
(3) 3 - 62	Prefrontal_R – Temporal_R	BA11 – BA41	(5.1,34.9,-17.4) – (39.9,-25.6,14.4)
(4) 3 - 173	Prefrontal_R – Parietal_L	BA11 – BA1	(5.1,34.9,-17.4) – (-41.2,-15.6,14.5)
(5) 4 - 180	Prefrontal_R – Parietal_L	BA11 – BA40	(15.6,34.1,-22.6) – (-42.2,-31.2,15.9)
(6) 17 - 268	Prefrontal_R – Brainstem_L	BA47 – n/a	(33.4,37.3,-16.4) – (-6.1,-18.9,-36.8)
(7) 24 - 172	MotorStrip_R – Parietal_L	BA6 – BA1	(6,-22.3,65.6) – (-23.5,-31.6,63.6)
(8) 26 - 77	MotorStrip_R – Occipital_R	BA6 – BA18	(26.3,-12.9,66.2) – (7.7,-75,25)
(9) 26 - 159	MotorStrip_R – MotorStrip_L	BA6 – BA6	(26.3,-12.9,66.2) – (-58.1,-5.6,27.2)
(10) 29 - 227	MotorStrip_R – Limbic_L	BA6 – BA30	(13.7,6.3,65.4) – (-7.5,-42.1,13.3)
(11) 33 - 77	MotorStrip_R – Occipital_R	BA1 – BA18	(42,-23.4,53.4) – (7.7,-75,25)
(12) 39 - 82	Parietal_R – Occipital_R	BA1 – BA17	(20,-33.2,69.8) – (14.6,-68.3,8.3)
(13) 39 - 211	Parietal_R – Occipital_L	BA1 – BA18	(20,-33.2,69.8) – (-8.9,-70.7,-1.7)
(14) 39 - 215	Parietal_R – Occipital_L	BA1 – BA17	(20,-33.2,69.8) – (-6,-81.2,12.2)
(15) 44 - 82	Parietal_R – Occipital_R	BA7 – BA17	(7.5,-57.3,61.8) – (14.6,-68.3,8.3)
(16) 48 - 131	Parietal_R – Brainstem_R	BA39 – n/a	(47.8,-61.6,34.7) – (6,-22.2,-42.3)
(17) 48 - 199	Parietal_R – Temporal_L	BA39 – BA37	(47.8,-61.6,34.7) – (-60.3,-50,-14)
(18) 49 - 104	Parietal_R – Cerebellum_R	BA39 – n/a	(41.4,-75.3,28) – (23.5,-35.9,-43)
(19) 49 - 237	Parietal_R – Cerebellum_L	BA39 – n/a	(41.4,-75.3,28) – (-8.7,-50.6,-39.6)
(20) 51 - 136	Temporal_R – Prefrontal_L	BA38 – BA11	(27.2,11.6,-39.2) – (-5.8,18.2,-21.6)
(21) 53 - 136	Temporal_R – Prefrontal_L	BA38 – BA11	(52.8,10.9,-21.8) – (-5.8,18.2,-21.6)

(22)	80 - 180	Occipital_R – Parietal_L	BA18 – BA40	(7.8,-88.6,11.9) – (-42.2,-31.2,15.9)
(23)	82 - 226	Occipital_R – Limbic_L	BA17 – BA31	(14.6,-68.3,8.3) – (-8.8,-42.6,50.1)
(24)	95 - 157	Limbic_R – Prefrontal_L	BA36 – BA6	(28,-28.4,-13.7) – (-46.2,7.9,28.6)
(25)	97 - 237	Limbic_R – Cerebellum_L	BA36 – n/a	(24.6,-2.6,-30.7) – (-8.7,-50.6,-39.6)
(26)	120 - 150	Subcortical_R – Prefrontal_L	n/a – BA8	(21.2,-36.4,22.6) – (-5,17.7,46.1)
(27)	122 - 169	Subcortical_R – Insula_L	BA48 – BA13	(13.7,-4.2,20.9) – (-38.7,8.1,-4.8)
(28)	124 - 128	Subcortical_R – Subcortical_R	BA49 – BA50	(26.6,6.3,0.1) – (5.5,-9.7,5.2)
(29)	124 - 258	Subcortical_R – Subcortical_L	BA49 – BA48	(26.6,6.3,0.1) – (-12.5,11.6,8.7)
(30)	125 - 185	Subcortical_R – Temporal_L	BA49 – BA38	(14,8.3,-9.5) – (-38,6.1,-37.9)
(31)	134 - 170	Prefrontal_L – Insula_L	BA11 – BA13	(-5.4,29.1,-10.1) – (-37.7,-12.9,-1.4)
(32)	138 - 169	Prefrontal_L – Insula_L	BA10 – BA13	(-6.9,48.3,-5.7) – (-38.7,8.1,-4.8)
(33)	213 - 235	Occipital_L – Limbic_L	BA18 – BA36	(-14.7,-84,-13.1) – (-21.4,-4.1,-29.4)
**Accordance negative network (ANN)**				
(1)	39 – 79	Parietal_R – Occipital_R	BA1 – BA18	(20,-33.2,69.8) – (7,-75.7,-2.8)
(2)	39 – 82	Parietal_R – Occipital_R	BA1 – BA17	(20,-33.2,69.8) – (14.6,-68.3,8.3)
(3)	39 – 215	Parietal_R – Occipital_L	BA1 – BA17	(20,-33.2,69.8) – (-6,-81.2,12.2)
(4)	160 – 215	MotorStrip _L – Occipital_L	BA6 – BA17	(-16.2,-19.2,69.5) – (-6,-81.2,12.2)
(5)	161 – 215	MotorStrip _L – Occipital_L	BA6 – BA17	(-6.5,-4.3,47.6) – (-6,-81.2,12.2)
(6)	172 – 208	Parietal_L –Occipital_L	BA1 – BA19	(-23.5,-31.6,63.6) – (-16.8,-84.9,33)
**Pearson's negative network (PNN)**				
(1)	1 – 197	Prefrontal_R – Temporal_L	BA11 – BA22	(13.9,56.8,-16.6) – (-57,-14.5,-6.9)
(2)	10 – 229	Prefrontal_R – Limbic_L	BA9 – BA54	(8.4,53.3,23.9) – (-21.5,-36.9,5.7)
(3)	16 – 142	Prefrontal_R –Prefrontal_L	BA47 – BA10	(53.6,24.8,0.9) – (-29.2,54.3,2.5)
(4)	24 – 172	MotorStrip_R – Parietal_L	BA6 – BA1	(6,-22.3,65.6) – (-23.5,-31.6,63.6)
(5)	25 – 215	MotorStrip_R –Occipital_L	BA6 – BA17	(7,-8.1,52.9) – (-6,-81.2,12.2)
(6)	39 – 82	Parietal_R – Occipital_R	BA1 – BA17	(20,-33.2,69.8) – (14.6,-68.3,8.3)

(7)	39 – 205	Parietal_R – Occipital_L	BA1 – BA19	(20,-33.2,69.8) – (-17,-50.7,0.8)
(8)	39 – 211	Parietal_R – Occipital_L	BA1 – BA18	(20,-33.2,69.8) – (-8.9,-70.7,-1.7)
(9)	39 – 215	Parietal_R – Occipital_L	BA1 – BA17	(20,-33.2,69.8) – (-6,-81.2,12.2)
(10)	48 – 131	Parietal_R – Brainstem_R	BA39 – n/a	(47.8,-61.6,34.7) – (6,-22.2,-42.3)
(11)	82 – 160	Occipital_R – MotorStrip _L	BA17 – BA6	(14.6,-68.3,8.3) – (-16.2,-19.2,69.5)
(12)	82 – 174	Occipital_R – Parietal_L	BA17 – BA7	(14.6,-68.3,8.3) – (-7.4,-34.1,67.5)
(13)	82 – 226	Occipital_R – Limbic_L	BA17 – BA31	(14.6,-68.3,8.3) – (-8.8,-42.6,50.1)
(14)	89 – 215	Limbic_R –Occipital_L	BA31 – BA17	(7.8,-23.1,44.9) – (-6,-81.2,12.2)
(15)	95 – 196	Limbic_R – Temporal_L	BA36 – BA20	(28,-28.4,-13.7) – (-51.8,-18.2,-28.8)
(16)	120 – 150	Subcortical_R – Prefrontal_L	n/a – BA8	(21.2,-36.4,22.6) – (-5,17.7,46.1)
(17)	124 – 128	Subcortical_R – Subcortical_R	BA49 – BA50	(26.6,6.3,0.1) – (5.5,-9.7,5.2)
(18)	134 – 170	Prefrontal_L – Insula_L	BA11 – BA13	(-5.4,29.1,-10.1) – (-37.7,-12.9,-1.4)
(19)	160 – 215	MotorStrip _L – Occipital_L	BA6 – BA17	(-16.2,-19.2,69.5) – (-6,-81.2,12.2)
(20)	161 – 215	MotorStrip _L – Occipital_L	BA6 – BA17	(-6.5,-4.3,47.6) – (-6,-81.2,12.2)
(21)	174 – 211	Parietal_L – Occipital_L	BA7 – BA18	(-7.4,-34.1,67.5) – (-8.9,-70.7,-1.7)
(22)	211 – 226	Occipital_L –Limbic_L	BA18 – BA31	(-8.9,-70.7,-1.7) – (-8.8,-42.6,50.1)

Notes: BA = Broadman area; L = left hemisphere; R = right hemisphere.

Supplementary Table 2. Detailed information about the primaty nodes of PAMS

Node index	Anatomical region	Broadman area	MNI coordinates			Node weight	Functional network
			x	y	z		
3	Medial Frontal Gyrus	11	5.1	34.9	–17.4	24.28	DM
49	Angular Gyrus	39	41.4	–75.3	28	22.69	DM
134	Medial Frontal Gyrus	25	–5.4	29.1	–10.1	14.13	DM
138	Middle Frontal Gyrus	10	–6.9	48.3	–5.7	13.29	DM
227	Posterior Cingulate	30	–7.5	–42.1	13.3	15.73	DM
1	Superior Frontal Gyrus	11	13.9	56.8	–16.6	11.65	FP
4	Inferior Frontal Gyrus	11	15.6	34.1	–22.6	11.78	FP
17	Inferior Frontal Gyrus	47	33.4	37.3	–16.4	12.57	FP
48	Inferior Parietal Lobule	39	47.8	–61.6	34.7	23.32	FP
157	Inferior Frontal Gyrus	6	–46.2	7.9	28.6	12.40	FP
24	Medial Frontal Gyrus	6	6	–22.3	65.6	8.18	MOT
26	Precentral Gyrus	6	26.3	–12.9	66.2	28.43	MOT
33	Postcentral Gyrus	4	42	–23.4	53.4	15.47	MOT

39	Postcentral Gyrus	3	20	–33.2	69.8	40.33	MOT
51	Superior Temporal Gyrus	38	27.2	11.6	–39.2	15.19	MOT
62	Insula	41	39.9	–25.6	14.4	13.53	MOT
159	Precentral Gyrus	6	–58.1	–5.6	27.2	12.29	MOT
170	Insula	13	–37.7	–12.9	–1.4	14.13	MOT
172	Postcentral Gyrus	3	–23.5	–31.6	63.6	8.18	MOT
173	Insula	13	–41.2	–15.6	14.5	10.75	MOT
180	Insula	40	–42.2	–31.2	15.9	38.55	MOT
197	Middle Temporal Gyrus	22	–57	–14.5	–6.9	11.65	MOT
2	Medial Frontal Gyrus	11	9.6	17.8	–19.5	13.60	SUB
29	Superior Frontal Gyrus	6	13.7	6.3	65.4	15.73	SUB
44	Postcentral Gyrus	7	7.5	–57.3	61.8	13.64	SUB
95	Parahippocampa Gyrus	36	28	–28.4	–13.7	12.40	SUB

122	Caudate	48	13.7	–4.2	20.9	13.31	SUB
124	Putamen	49	14	8.3	–9.5	22.13	SUB
128	Thalamu	50	5.5	–9.7	5.2	12.64	SUB
131	Brainstem	/	6	–22.2	–42.3	13.32	SUB
136	Medial Frontal Gyrus	11	–5.8	18.2	–21.6	24.50	SUB
169	Insula	13	–38.7	8.1	–4.8	26.60	SUB
226	Precuneus	31	–8.8	–42.6	50.1	14.53	SUB
258	Caudate	48	–10.4	10.9	–8.1	9.48	SUB
268	Brainstem	/	–6.1	–18.9	–36.8	12.57	SUB
77	Cuneus	18	7.7	–75	25	31.60	VI
80	Cuneus	18	7.8	–8.6	11.9	13.17	VI
82	Calcarine	17	14.6	–68.3	8.3	42.36	VI
211	Lingual Gyrus	18	–8.9	–70.7	–1.7	13.72	VI
215	Calcarine	17	–6	–81.2	12.2	12.42	VI
104	Cerebellum Posterior Lobe	/	23.5	–35.9	–43	10.89	CER
237	Cerebellum Posterior Lobe	/	–8.7	–50.6	–39.6	21.01	CER

Supplementary Table 3. Regression coefficients of the primary connectivity in PAMS

Edge index	Regression coefficient	Network interaction
180 - 80	13.16778	MOT-VI
26 – 77	16.13416	MOT-VI
33 - 77	15.46579	MOT-VI
39 - 82	14.1919	MOT-VI
39 - 211	13.71739	MOT-VI
39 - 215	12.42112	MOT-VI
62 - 3	13.52901	MOT-DM
173 - 3	10.74906	MOT-DM
170 - 134	14.13092	MOT-DM
95 -157	12.3993	SUB-FP
268 - 17	12.57169	SUB-FP
131 - 48	13.32296	SUB-FP
169 - 122	13.30889	SUB-SUB
128 - 124	12.64405	SUB-SUB
258 - 124	9.481912	SUB-SUB
29 - 227	15.73335	SUB-DM
169 - 138	13.28804	SUB-DM
2 - 180	13.60165	SUB-MOT
136 - 51	15.19136	SUB-MOT
226 - 82	14.52914	SUB-VI
44 - 82	13.63762	SUB-VI
197 - 1	11.65373	MOT-FP
180 - 4	11.77891	MOT-FP
104 - 49	10.89276	CER-DM
237 - 49	11.80331	CER-DM
172 - 24	8.176035	MOT-MOT
159 - 26	12.29537	MOT-MOT

Supplementary Figures：

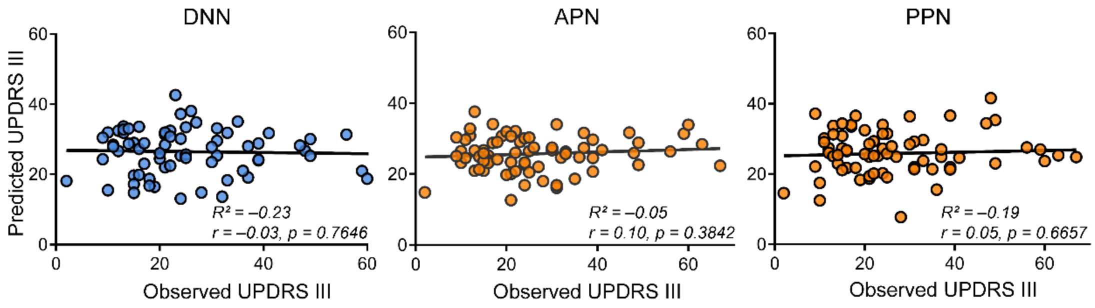

Supplementary Figure 1. The predictive performance of abandoned models in Study 1. The discordance negative network (DNN), accordance positive network (APN), and Pearson's positive network (PPN) showed less accuracy to predict the UPDRS III scores comparing with their opposite modes (DPN, ANN, and PNN). The cold color (blue) indicates the negative correlation between functional interactions and UPDRS-III scores, while the warm color (orange) stands for the positive correlation. DPN = Discordance positive network; ANN = Accordance negative network; PNN = Pearson's negative network.

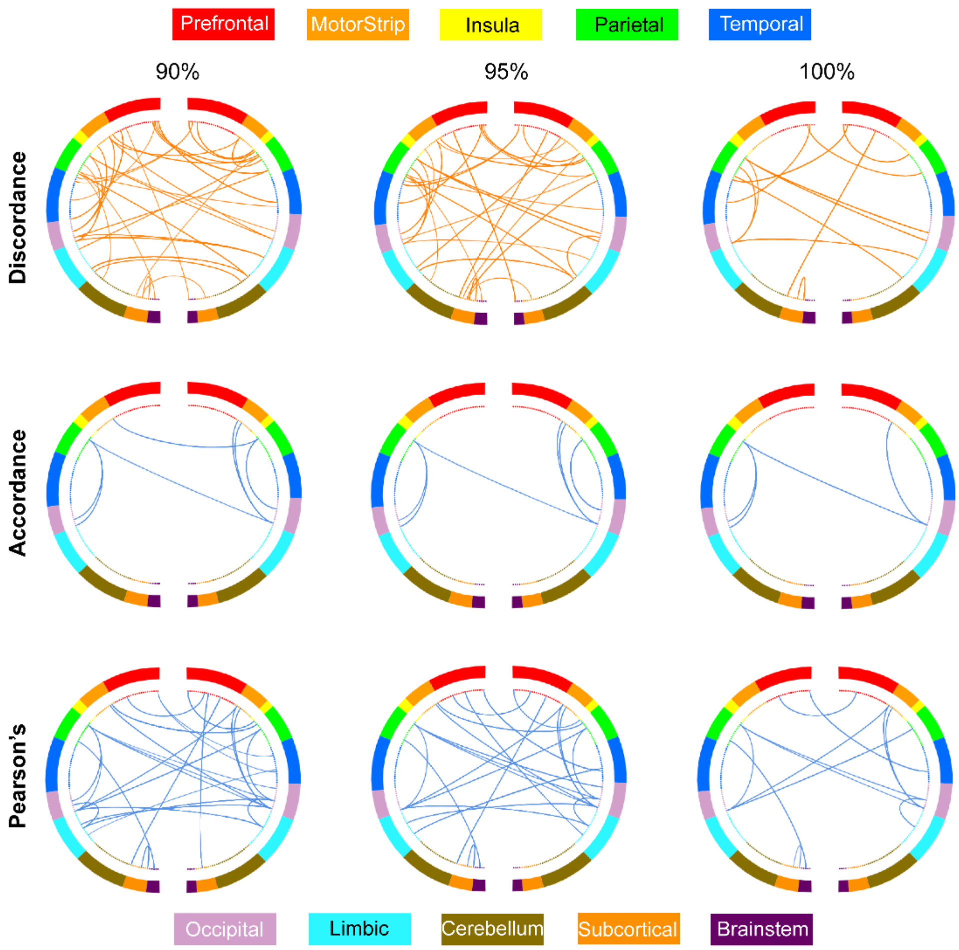

Supplementary Figure 2. Predictive networks of different thresholds. We showed the predictive networks of 90%, 95% and 100% thresholds using the chord chart in the Bioimage Suite (https://bioimagesuiteweb.github.io/webapp/).

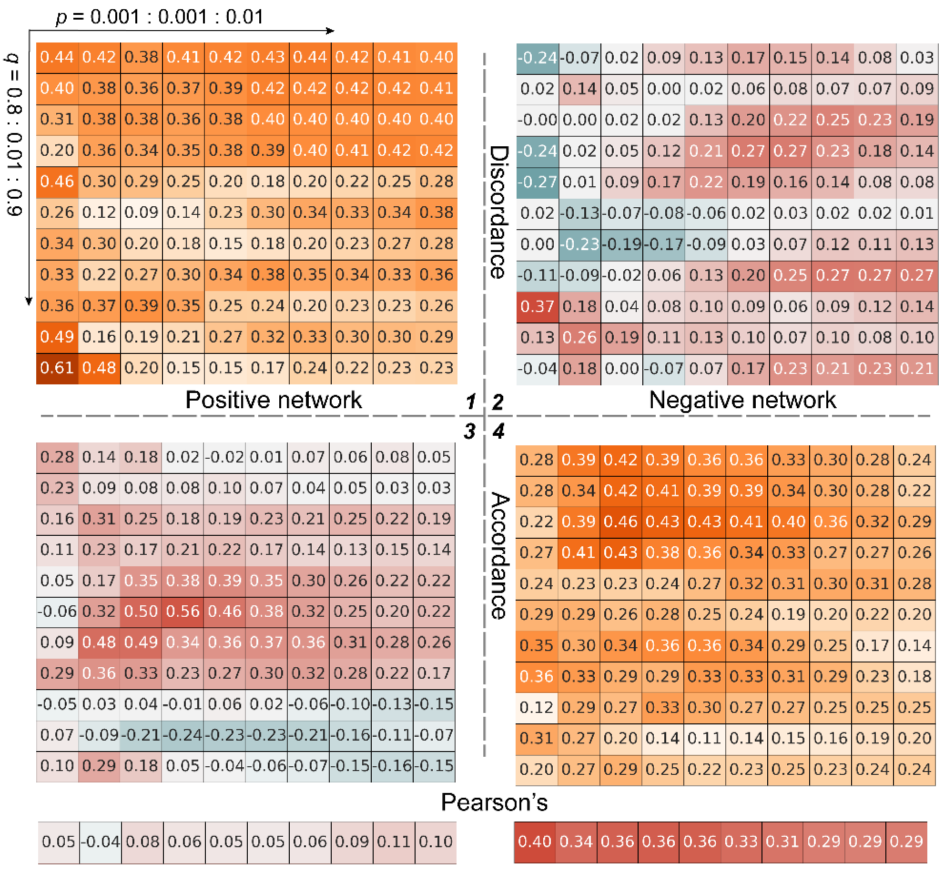

Supplementary Figure 3. The predictive performance with multiple parameters. Given that there are two important parameters when constructing connectome-models (p: the threshold for feature selection, q: the threshold for binarizing time courses), we showed the correlation *r* values of the combinations of p (from 0.001 to 0.01 with a step of 0.001) and q (from 0.8 to 0.9 with a step of 0.1) for each measure. Positive network means the functional connectivity was positively correlated with UPDRS-III scores, and negative network means the functional connectivity was negatively correlated with UPDRS-III scores.

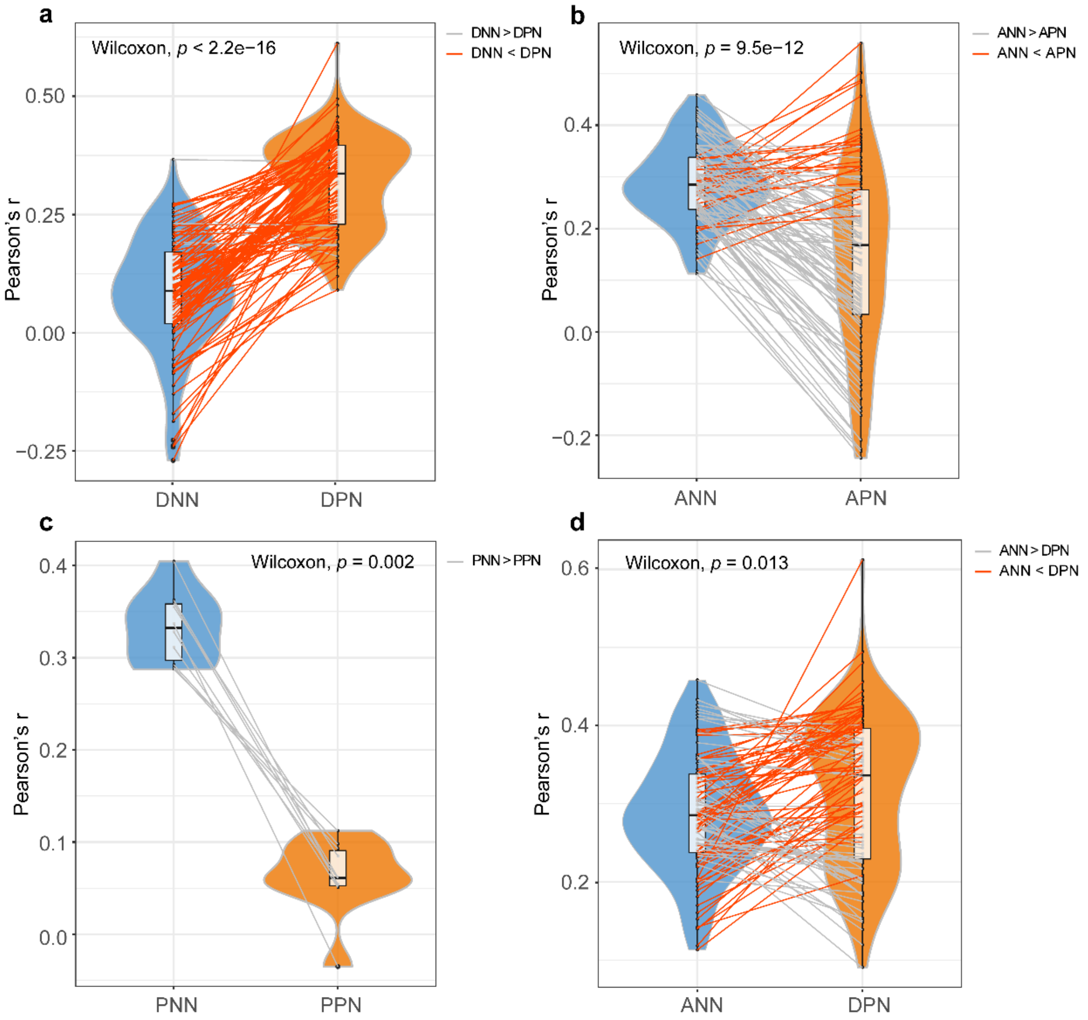

Supplementary Figure 4. Comparisons of the predictive performance on different models. There were 110 combinations of p (10 choices) and q (11 choices), and we performed the paired sample Wilcoxon test on all Pearson's r values between the observed and predicted motor scores without assuming the normal distribution. We found significant differences between each pair, in particular the opposite models of each measure. Boxplots depict the median (the thick black line), upper and lower quartiles (box), 1.5 times interquartile range (whiskers) of the Pearson's r values. DPN = Discordance positive network; DNN = Discordance negative network; APN = Accordance positive network ANN = Accordance negative network; PPN = Pearson's positive network; PNN = Pearson's negative network.

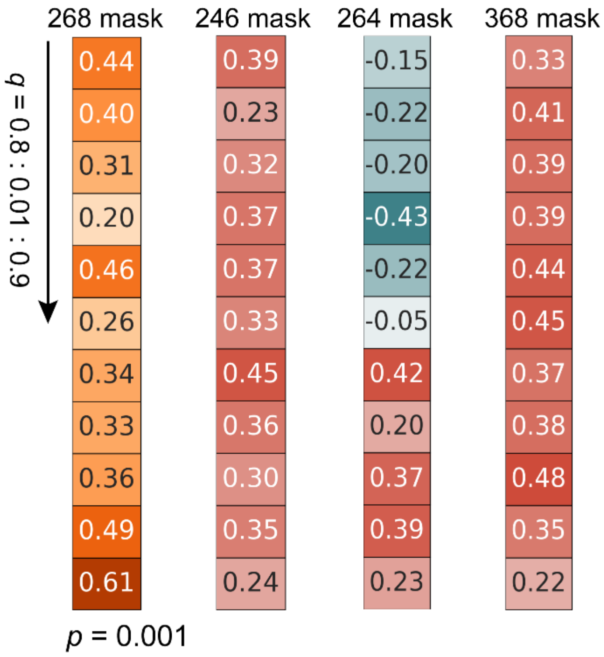

Supplementary Figure 5. Retest predictive performance on different functional masks. We examined the discordance connectivity-based predictive performance on Fan-246 atlas [1], Power-264 atlas [2], and Shen-368 atlas [3]. The threshold of feature selection (*p*) was set to 0.001, and the threshold of time courses encoding (*q*) was from 0.8 to 0.9.

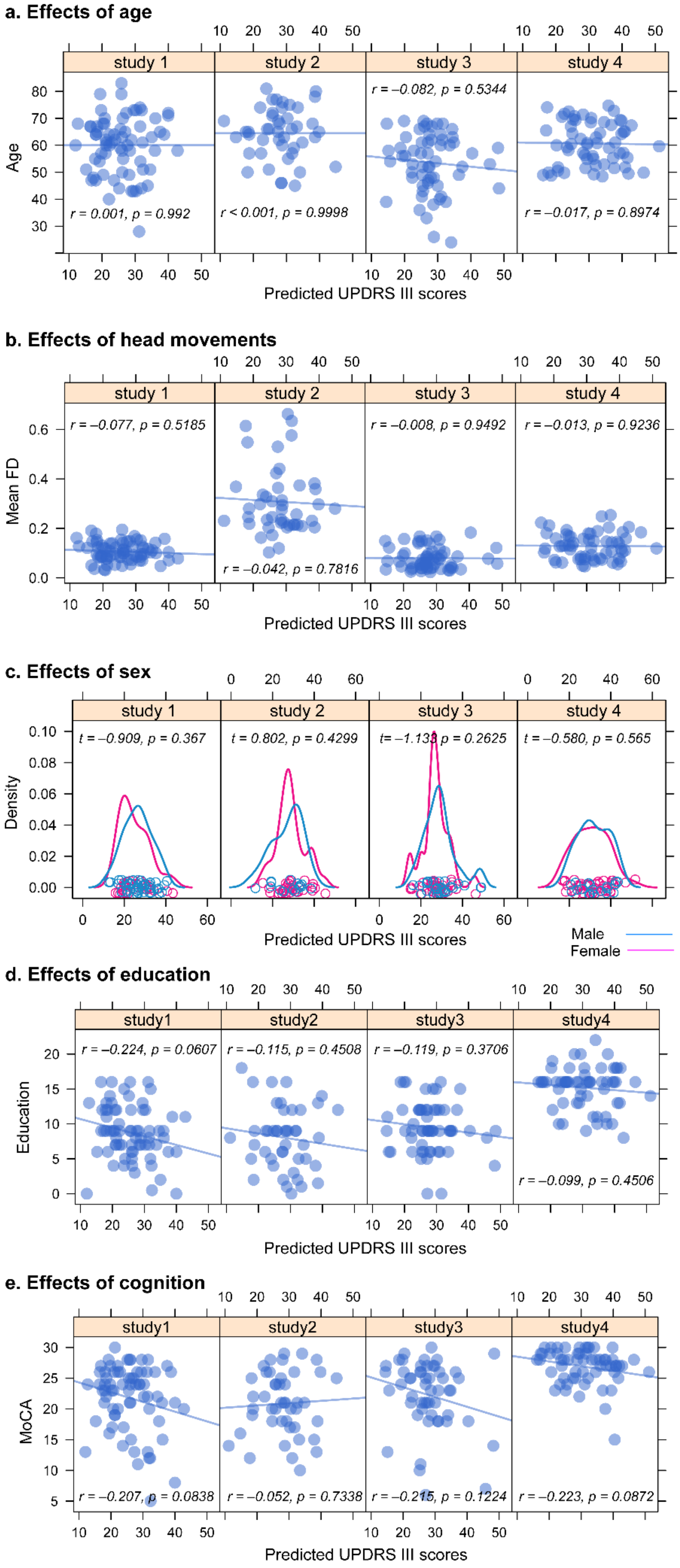
a. Effects of age
study 1
study 2
study 3
study 4
Age
r = 0.001, p = 0.992
r < 0.001, p = 0.9998
r = –0.082, p = 0.5344
r = –0.017, p = 0.8974
Predicted UPDRS III scores
b. Effects of head movements
study 1
study 2
study 3
study 4
Mean FD
r = –0.077, p = 0.5185
r = –0.042, p = 0.7816
r = –0.008, p = 0.9492
r = –0.013, p = 0.9236
Predicted UPDRS III scores
c. Effects of sex
study 1
study 2
study 3
study 4
Density
t = –0.909, p = 0.367
t = 0.802, p = 0.4299
t= –1.133, p = 0.2625
t = –0.580, p = 0.565
Predicted UPDRS III scores
Male
Female
d. Effects of education
study1
study2
study3
study4
Education
r = –0.224, p = 0.0607
r = –0.115, p = 0.4508
r = –0.119, p = 0.3706
r = –0.099, p = 0.4506
Predicted UPDRS III scores
e. Effects of cognition
study1
study2
study3
study4
MoCA
r = –0.207, p = 0.0838
r = –0.052, p = 0.7338
r = –0.215, p = 0.1224
r = –0.223, p = 0.0872
Predicted UPDRS III scores

Supplementary Figure 6. Confounding effects analysis. The effects of age, head motion, sex education, and cognition were considered in the predictive model. We investigated the Pearson's correlation between predicted scores and ages (a), mean FD (b), years of education (d), as well as MoCA scores (e). Two sample t test was performed between males and females (c). FD = Framewise displacement, MoCA = Montreal Cognitive Assessment.

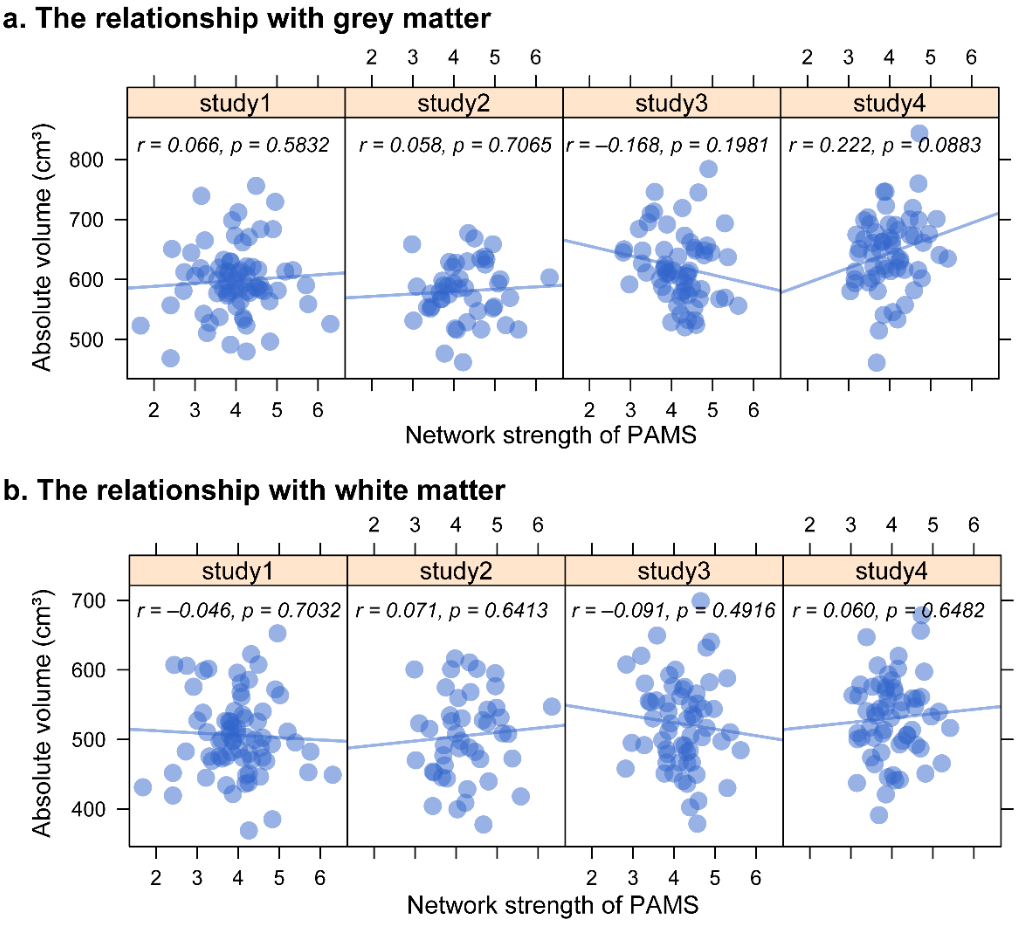

Supplementary Figure 7. The dependency between structural measures and functional network strength. We calculated absolute volumes of grey matter (GM) and white matter (WM), and investigated the relationship between the network strength of PAMS (the sum of all antagonistic interactions) and the volumes of GM (a), and WM (b) using Pearson's correlation. PAMS = Parkinson's antagonistic motor signature.

Supplementary References:

1. Fan, L., et al., *The Human Brainnetome Atlas: A New Brain Atlas Based on Connectional Architecture.* Cereb Cortex, 2016. **26**(8): p. 3508-26.
2. Power, J.D., et al., *Functional network organization of the human brain.* Neuron, 2011. **72**(4): p. 665-78.
3. Salehi, M., et al., *There is no single functional atlas even for a single individual: Functional parcel definitions change with task.* Neuroimage, 2020. **208**: p. 116366.
