# Supplementary material for: Antagonistic network signature of motor function in Parkinson’s disease revealed by connectome-based predictive modeling
Source: NPJ Parkinsons Dis. 2022 Apr 22;8:49. doi: 10.1038/s41531-022-00315-w (PMC9033778; doi:10.1038/s41531-022-00315-w)
Supplement: Supplementary file 2 — Reporting Summary [file 41531_2022_315_MOESM2_ESM.pdf]

nature portfolio

Corresponding author(s): Rong Li, Xiaofei Hu, Li Meng, Huafu Chen

Last updated by author(s): Mar 19, 2022

Reporting Summary

Nature Portfolio wishes to improve the reproducibility of the work that we publish. This form provides structure for consistency and transparency in reporting. For further information on Nature Portfolio policies, see our Editorial Policies and the Editorial Policy Checklist.

Statistics

For all statistical analyses, confirm that the following items are present in the figure legend, table legend, main text, or Methods section.

n/a	Confirmed	
☐	☒	The exact sample size (n) for each experimental group/condition, given as a discrete number and unit of measurement
☐	☒	A statement on whether measurements were taken from distinct samples or whether the same sample was measured repeatedly
☐	☒	The statistical test(s) used AND whether they are one- or two-sided<br>*Only common tests should be described solely by name; describe more complex techniques in the Methods section.*
☐	☒	A description of all covariates tested
☐	☒	A description of any assumptions or corrections, such as tests of normality and adjustment for multiple comparisons
☐	☒	A full description of the statistical parameters including central tendency (e.g. means) or other basic estimates (e.g. regression coefficient) AND variation (e.g. standard deviation) or associated estimates of uncertainty (e.g. confidence intervals)
☐	☒	For null hypothesis testing, the test statistic (e.g. F, t, r) with confidence intervals, effect sizes, degrees of freedom and P value noted<br>*Give P values as exact values whenever suitable.*
☒	☐	For Bayesian analysis, information on the choice of priors and Markov chain Monte Carlo settings
☐	☒	For hierarchical and complex designs, identification of the appropriate level for tests and full reporting of outcomes
☐	☒	Estimates of effect sizes (e.g. Cohen's d, Pearson's r), indicating how they were calculated

Our web collection on statistics for biologists contains articles on many of the points above.

Software and code

Policy information about availability of computer code

Data collection	No software was used.
Data analysis	Data analysis was performed with Data Processing Assistant for Resting-State fMRI (DPARSF v5.0), Statistical Parametric Mapping (SPM12), Matlab2018b, and RStudio (R version 3.6.3). Relevant codes were deposited in Giithub (https://github.com/XuyangWang96/PAMS).

For manuscripts utilizing custom algorithms or software that are central to the research but not yet described in published literature, software must be made available to editors and reviewers. We strongly encourage code deposition in a community repository (e.g. GitHub). See the Nature Portfolio guidelines for submitting code & software for further information.

Data

Policy information about availability of data

All manuscripts must include a data availability statement. This statement should provide the following information, where applicable:

- Accession codes, unique identifiers, or web links for publicly available datasets
- A description of any restrictions on data availability
- For clinical datasets or third party data, please ensure that the statement adheres to our policy

Data for the PPMI are freely available in the public domain through the Parkinson's Progression Markers Initiative website (PPMI, https://www.ppmi-info.org). Data for the TMMU and CSU are available from the corresponding author subject to anonymization to protect privacy of clinical data and implementation of a data sharing agreement as required by the local IRB.

Field-specific reporting

Please select the one below that is the best fit for your research. If you are not sure, read the appropriate sections before making your selection.

☒ Life sciences ☐ Behavioural & social sciences ☐ Ecological, evolutionary & environmental sciences

For a reference copy of the document with all sections, see nature.com/documents/nr-reporting-summary-flat.pdf

Life sciences study design

All studies must disclose on these points even when the disclosure is negative.

Sample size	According to the paper "Sample size requirements for estimating Pearson, Kendall and Spearman correlations" (2000), a sample size for running Pearson's correlation should be equal or superior to 25. Thus for each study we recruited sufficient samples to make results credible (Study 1: n = 71; Study 2: n = 45; Study 3: n = 60; Study 4: n = 60), and the total number of subjects is 236.
Data exclusions	Our exclusions include: 1. Patients without any other neurological or psychiatric disorders (such as seizures, stroke, severe depression, or claustrophobia). 2. Poor image quality. 3. Functional scanning with large head movements.
Replication	Independent samples were collected from different center to test the predictive model which was established in the discovery cohort.
Randomization	This is not relevant to our study, because we aimed to explore the mapping between the brain and motor function in patients with Parkinson's disease (in fact only one group determined by neurological doctors).
Blinding	This is not relevant to our study, because we only recruited patients with Parkinson's disease and did not allocate them into subgroups.

Reporting for specific materials, systems and methods

We require information from authors about some types of materials, experimental systems and methods used in many studies. Here, indicate whether each material, system or method listed is relevant to your study. If you are not sure if a list item applies to your research, read the appropriate section before selecting a response.

Materials & experimental systems

n/a	Involved in the study	
☒	☐	Antibodies
☒	☐	Eukaryotic cell lines
☒	☐	Palaeontology and archaeology
☒	☐	Animals and other organisms
☐	☒	Human research participants
☒	☐	Clinical data
☒	☐	Dual use research of concern

Methods

n/a	Involved in the study	
☒	☐	ChIP-seq
☒	☐	Flow cytometry
☐	☒	MRI-based neuroimaging

Human research participants

Policy information about studies involving human research participants

Population characteristics	In this research, we collected demographical and clinical variables including age, gender, years of education, cognitive scores, head movements, and levodopa equivalent daily dose as covariates (details in Table 1).
Recruitment	Patients with Parkinson's disease were recruited based on the diagnosis from experienced neurological doctors.
Ethics oversight	the Southwest Hospital of the Third Military Medical University; the Xiangya Hospital of Central South University; the Parkinson's Progression Markers Initiative

Note that full information on the approval of the study protocol must also be provided in the manuscript.

Magnetic resonance imaging

Experimental design

Design type	resting state
Design specifications	In Study 1 and Study 2, the scanning of resting state lasted for 8 minutes. In Study 3, the scanning lasted for 6 minutes. In Study 4, the scanning lasted for 8 minutes and 24 seconds.

Behavioral performance measures: No Behavioral tasks

Acquisition

Imaging type(s): functional, structural

Field strength: 3T

Sequence & imaging parameters: Site I: This cohort was recruited from the TMMU, with a total of 116 patients with PD (n = 71 in Study 1 and n = 45 in Study 2). On a 3.0 T Siemens Trio Total imaging matrix (Tim) whole-body MRI system (Siemens Medical Solutions, Erlangen, Germany), functional data were collected transversely by using an echo-planar imaging (EPI) sequence with the following setting: TR = 2000 ms, TE = 30 ms, flip angle = 90°, FOV = 192 mm×192 mm, slice thickness = 3 mm, voxel size = 3.0 mm×3.0 mm×3.99 mm, and 36 slices. For each subject, a total of 240 volumes were obtained from the scan time of 8 min. Structural 3D T1-weighted images were acquired using a magnetization-prepared rapid gradient-echo (MP-RAGE) sequence for co-registration with functional images: TR = 1900 ms, TE = 2.52 ms, flip angle = 9°, slice thickness = 1 mm, slices = 176, FOV = 256 mm×256 mm, matrix size = 256×256, and voxel size = 1 mm×1 mm×1 mm.
Site II: Patients with PD in the CSU sample (n = 60 for Study 3) were scanned on a 3.0 T GE Signa MR system (General Electric, Fairfield, CT, USA). Functional data were acquired using a gradient echo EPI sequence with the following parameters: TR = 2000 ms, TE = 30 ms, flip angle = 90°, FOV = 220 mm×220 mm, slice thickness = 4.0 mm, voxel size = 3.44 mm×3.44 mm×4.60 mm, and 32 slices. Finally, 180 volumes were acquired for each subject over 6 min. T1 images were also collected for normalization: TR = 7.792 ms, TE = 2.984 ms, flip angle = 7°, slice thickness = 1 mm, slices = 188, matrix size = 256×256, and voxel size = 1 mm×1 mm×1 mm.
PPMI: All patients (n = 60 for Study 4) were scanned on a 3.0 T Siemens MRI system using the same sequence as Site I, and the parameters for functional images are as follows: TR = 2400 ms, TE = 25 ms, flip angle = 80°, FOV = 224 mm×217 mm, slice thickness = 3.3 mm, voxel size = 3.29 mm×3.29 mm×3.30 mm, and 40 slices. A total of 210 images were acquired for each participant in 8 min. The scanning parameters for T1 images are as follows: TR = 2300 ms, TE = 2.98 ms, flip angle = 9°, slice thickness = 1 mm, slices = 176, FOV = 240 mm×256 mm, matrix size = 240×256, and voxel size = 1 mm×1 mm×1 mm.

Area of acquisition: A whole brain scan was used.

Diffusion MRI: ☐ Used ☒ Not used

Preprocessing

Preprocessing software: Data Processing Assistant for Resting-State fMRI (DPARSF v5.0), Statistical Parametric Mapping (SPM12)

Normalization: The functional data was normalized by DARTEL method (a nonlinear approach.)

Normalization template: Dartel template of each subject was obtained for further normalization to Montreal Neurological Institute space.

Noise and artifact removal: Regression Covariates included the signals from white matter and cerebrospinal fluid, Friston-24 head motion parameters (six motion parameters, six temporal derivatives, and their corresponding squares), and the whole brain were regressed in the native image space. After normalization, residual images were temporally smoothed with a band-pass filter (0.01–0.1Hz) to retain the most neurological-related signals.

Volume censoring: Framewise displacement (FD) was estimated by Jekinson's method (calculated in DPARSF v5.0). The scanning with head translation > 3 mm, head rotation > 3°, and mean FD> 0.2 mm was abandoned for establish predictive modeling and validations.

Statistical modeling & inference

Model type and settings: We used the connectome-based predictive modeling (CPM) in this reaesrch

Effect(s) tested: N/A

Specify type of analysis: ☐ Whole brain ☒ ROI-based ☐ Both

Anatomical location(s): We used Shen-268 brain mask to define the regions of interests in this study.

Statistic type for inference (See Eklund et al. 2016): N/A

Correction: Permutation test was used in this reaearch.

Models & analysis

n/a	Involved in the study
☐	☒ Functional and/or effective connectivity
☒	☐ Graph analysis
☐	☒ Multivariate modeling or predictive analysis

Functional and/or effective connectivity	We use three measures of functional connectivity: Pearson's correlation, accordance, discordance
Multivariate modeling and predictive analysis	Functional connectivity served as independent variable here. For feature selection, partial correlation was used between each connectivity and motor scores (age as a confounder). A threshold of $P < 0.001$ was set to reduce dimensions. For model building, partial least square regression (PLSR) was adopted to learn the regression line and provide predictive weights on each feature. The predictive performance was evaluated with a leave-one-out cross-validation method on the discovery cohort, which reduced the risk of overfitting by removing one subject from training data and building a predictive model on N–1 subjects.
